# Accumulation of Multipotent Hematopoietic Progenitors in Peripheral Lymphoid Organs of Mice Over-expressing Interleukin-7 and Flt3-Ligand

**DOI:** 10.3389/fimmu.2018.02258

**Published:** 2018-10-10

**Authors:** Fabian Klein, Lilly von Muenchow, Giuseppina Capoferri, Stefan Heiler, Llucia Alberti-Servera, Hannie Rolink, Corinne Engdahl, Michael Rolink, Mladen Mitrovic, Grozdan Cvijetic, Jan Andersson, Rhodri Ceredig, Panagiotis Tsapogas, Antonius Rolink

**Affiliations:** ^1^Department of Biomedicine, Developmental and Molecular Immunology, University of Basel, Basel, Switzerland; ^2^Discipline of Physiology, College of Medicine & Nursing Health Science, National University of Ireland, Galway, Ireland

**Keywords:** IL-7, Flt3-ligand, hematopoiesis, cytokines, B cell development

## Abstract

Interleukin-7 (IL-7) and Flt3-ligand (FL) are two cytokines important for the generation of B cells, as manifested by the impaired B cell development in mice deficient for either cytokine or their respective receptors and by the complete block in B cell differentiation in the absence of both cytokines. IL-7 is an important survival and proliferation factor for B cell progenitors, whereas FL acts on several early developmental stages, prior to B cell commitment. We have generated mice constitutively over-expressing both IL-7 and FL. These double transgenic mice develop splenomegaly and lymphadenopathy characterized by tremendously enlarged lymph nodes even in young animals. Lymphoid, myeloid and dendritic cell numbers are increased compared to mice over-expressing either of the two cytokines alone and the effect on their expansion is synergistic, rather than additive. B cell progenitors, early progenitors with myeloid and lymphoid potential (EPLM), common lymphoid progenitors (CLP) and lineage^−^, Sca1^+^, kit^+^ (LSK) cells are all increased not only in the bone marrow but also in peripheral blood, spleen and even lymph nodes. When transplanted into irradiated wild-type mice, lymph node cells show long-term multilineage reconstitution, further confirming the presence of functional hematopoietic progenitors therein. Our double transgenic mouse model shows that sustained and combined over-expression of IL-7 and FL leads to a massive expansion of most bone marrow hematopoietic progenitors and to their associated presence in peripheral lymphoid organs where they reside and potentially differentiate further, thus leading to the synergistic increase in mature lymphoid and myeloid cell numbers. The present study provides further *in vivo* evidence for the concerted action of IL-7 and FL on lymphopoiesis and suggests that extramedullary niches, including those in lymph nodes, can support the survival and maintenance of hematopoietic progenitors that under physiological conditions develop exclusively in the bone marrow.

## Introduction

Cytokines are important regulators for the development and function of immune cells. Apart from influencing the survival, expansion and effector function of mature immune cells in peripheral lymphoid organs, cytokines also have a crucial role in the continuous generation of all blood cell lineages (hematopoiesis), which occurs in the bone marrow and thymus. During hematopoiesis cytokines can influence the lineage output of hematopoietic progenitors by selectively promoting their survival, proliferation or developmental potential (1–3). Two of the most important cytokines for the generation of lymphoid cells are Flt3-ligand (FL) and Interleukin-7 (IL-7).

FL is produced by many cell types, but within the hematopoietic system it acts mainly on early, multipotent progenitors, which are the ones expressing its receptor, CD135 (Flt3) (4–6). CD135 is the only known receptor for FL and belongs to the type-III tyrosine kinase receptor family, which also includes CD117 (kit) and platelet-derived growth factor receptor (PDGF-R). CD135 expression within the lineage^−^, Sca1^+^, kit^+^ (LSK) compartment of hematopoietic progenitors is associated with loss of self-renewal capacity and preservation of multilineage developmental potential (7). Oligopotent myeloid and lymphoid progenitors retain CD135 expression until they become committed to their respective lineages. From that point on, they down-regulate expression of the receptor, with the exception of dendritic cells (DC), which remain CD135^+^ after maturation. The importance of FL in B cell development is manifested by the reduced capacity of *Flt3l*^−/−^ and *Flt3*^−/−^ hematopoietic progenitors to reconstitute the B cell compartment of lymphopenic mice (8, 9). In addition, B cell regeneration after irradiation or chemically induced myeloablation is dependent on FL (10). Since CD135 is down-regulated in committed B cell progenitors after Pax5 expression (11), the effect of FL signaling on B cell development is attributed to its role in maintaining normal numbers of oligopotent CD135^+^ common lymphoid progenitors (CLP) and early progenitors with lymphoid and myeloid potential (EPLM) (12, 13).

CLP are phenotypically defined by the expression of CD127, the α-subunit of the receptor for IL-7 (14). CD127 expression is initiated at the CLP stage and remains expressed throughout the early stages of B cell development until the progenitors start to rearrange their light chain immunoglobulin genes (small pre-B stage). IL-7 was initially identified as a growth factor for B cells (15) and its essential role in lymphoid development has been proven both by its ability to maintain and expand lymphoid cells *in vitro* (16) and by the severe defect in B and T cell development observed in *Il7*^−/−^ and *Il7r*α^−/−^ mice (17, 18). Early T cell progenitors require IL-7 mainly for their survival, since over-expression of the anti-apoptotic protein Bcl2 can significantly rescue T cell development in *Il7r*α^−/−^ mice (19, 20). IL-7 is also important for the survival and homeostatic expansion of mature T cells in the periphery (21). The fact that B cell development in *Il7r*α^−/−^ mice cannot be rescued by Bcl2 over-expression (22, 23), together with the absence of early B-cell factor 1 (Ef1) expression in *Il7r*α^−/−^ CLP (24) has led to the hypothesis that by initiating Ebf1 expression, IL-7 might act as an instructive cytokine for B cell commitment. However, this could also be explained by a survival role of IL-7 on CLP, since the Ebf1-expressing Ly6D^+^ compartment of CLP is severely reduced in *Il7*^−/−^ mice (25). Furthermore, B cell commitment in the absence of IL-7 signaling can be restored by over-expressing Bcl2 in mice lacking the IL-7 signaling mediator STAT5 (26) and by over-expressing FL in *Il7*^−/−^ mice (13), therefore indicating that the role of this cytokine in commitment of progenitors to the B cell lineage is permissive, rather than instructive. Following Pax5 expression and lineage commitment, B cell progenitors require IL-7 for their survival and expansion. This has been clearly manifested in mice expressing high, sustained levels of IL-7, which resulted in expansion of pre-B cell progenitors in the bone marrow and in some cases in the development of B cell tumors (27–29). This increase in pro-B and pre-B cell numbers resulted in their accumulation in secondary lymphoid organs, such as spleen and lymph nodes.

As evident from the above, both FL and IL-7 are pivotal for the generation of normal B cell numbers, a fact highlighted by the complete absence of B cells in mice lacking both cytokines (30). Their combined effect is mostly exerted at different stages of B cell development, with FL being crucial for the generation of early multipotent progenitors and IL-7 for their survival and expansion after B cell commitment (13). However, they also act synergistically at the CLP/EPLM stage, where the receptors for both cytokines are simultaneously expressed, possibly through activation of separate signaling pathways (31). We have previously generated mice expressing high, sustained levels of human FL (hereafter FLtg) (32), which, when crossed to *Il7*^−/−^ mice, showed a complete rescue of Ly6D^+^ CLP/EPLM numbers (13). Interestingly, however, we have observed that mice over-expressing FL have reduced pre-B cell numbers compared to their wild-type (WT) counterparts (32). This could not be a direct effect of FL on pre-B cells, as they do not express CD135. Since pre-B cells are highly dependent on IL-7 for their expansion, we hypothesized that IL-7 availability might be reduced in FLtg bone marrow, due to the high number of CD127^+^ CLP and EPLM progenitors, which might consume a large part of the available IL-7. Binding of IL-7 to its receptor on CD127^+^ target cells has been proposed as a mechanism that regulates the abundance of the cytokine in different tissues (33).

In order to test this hypothesis, and to further study the synergy between FL and IL-7 in promoting lymphoid development *in vivo*, we bred FLtg mice with transgenic mice expressing high amounts of IL-7 (hereafter IL7tg) and analyzed the F_1_ generation. Double transgenic mice (hereafter FLtgxIL7tg) had a phenotype that combined features of the single transgenic phenotypes and in terms of expansion of lymphoid cells, revealed synergy between the two cytokines. Thus, double transgenic mice exhibited splenomegaly and abnormally enlarged lymph nodes (LN), in which B and T cell numbers were increased more than in the single transgenic mice. Moreover, large numbers of B cell progenitors as well as CD19^−^ multipotent progenitors were found in the LN of FLtgxIL7tg mice. Transplantation of LN FLtgxIL7tg cells into myelo-ablated recipients showed that they contained hematopoietic progenitors with long-term multilineage developmental potential, suggesting that the LN niche can support the survival and maintenance of early hematopoietic progenitors.

## Materials and methods

### Mice

C57BL/6 (CD45.1^+^ and CD45.2^+^), FLtg, IL7tg, FLtgxIL7tg and NOD/SCID/*Il2r*γ^−/−^ mice were bred and maintained in our animal facility unit under specific pathogen-free conditions. All mice used were 5–9 weeks old. All animal experiments were carried out within institutional guidelines (authorization numbers 1886 and 1888 from cantonal veterinarian office, Canton Basel-Stadt).

### Antibodies, flow cytometry and sorting

For analysis, cells were flushed from femurs and tibias of the two hind legs of mice or single-cell suspensions of spleen and lymph node (inguinal and axillary) cells were made. For blood analysis, blood was taken from the heart of euthanized animals or from the tail vein of live ones and white blood cells were isolated after separation with Ficoll. Stainings were performed in PBS containing 0.5% BSA and 5 mM EDTA. For intra-cellular Foxp3 staining, cells were fixed and permeabilized after cell-surface staining using the Foxp3 Fix/Perm buffer set (eBioscience), and subsequently stained with PE-conjugated anti-Foxp3. The following antibodies were used for flow cytometry (from BD Pharmingen, eBioscience, BioLegend, or produced in house): anti-B220 (RA3-6B2), anti-CD117 (2B8), anti-CD19 (1D3), anti-NK1.1 (PK136), anti-SiglecH (551), anti-CD11c (HL3), anti-Ly6D (49-H4), anti-CD127 (SB/199), anti-Sca1 (D7), anti-IgM (M41), anti-Foxp3 (FJK-16s), anti-CD4 (GK1.5), anti-CD8 (53.6.7), anti-Gr1 (RB6-8C5), anti-CD11b (M1.7015), anti-MHC-II (M5/114.15.2), anti-XCR1 (ZET), anti-CD93 (PB493), anti-CD48 (HM48-1), anti-CD150 (TC15-12F12.2), anti-Ter119 (TER-119), anti-CD3 (145-2C11), anti-CD41 (MWReg30), anti-CD105 (MJ7/18), anti-CD16/32 (2.4G2), anti-S1PR1 (713412), anti-CD44 (IM7), anti-CXCR4 (L276F12), anti-CD5 (53-7.3), and anti-Ki67 (B56). Lineage cocktail included antibodies against: CD4, CD8, CD11b, CD11c, Gr1, B220, CD19, Ter119, and NK1.1. Flow cytometry was done using a BD LSRFortessa (BD Biosciences) and data were analyzed using FlowJo Software (Treestar). For cell sorting, a FACSAria IIu (BD Biosciences) was used (>98% purity).

### Flt3-ligand and IL-7 quantification

Sera were collected from mice of all genotypes and ELISA was performed using the Invitrogen human Flt3-ligand and mouse IL-7 ELISA kits, following the provider's instructions.

### Quantitative PCR

Spleens were homogenized using the FastPrep^®;^ homogenizer (MP Biomedicals) and RNA was extracted with Trizol (Invitrogen) following the provider's protocol. Five hundred micrograms of total RNA was used to synthesize cDNA using the GoScript reverse transcriptase (Promega). Quantitative PCR for the detection of *Il7* transcripts was performed using SYBR™ Green (Promega). Primers used: *Hprt*-Forw: atcagtcaacgggggacataaa; *Hprt*-Rev: tggggctgtactgcttaacca; *Il7*-Forw: GATAGTAATTGCCCGAATAATGAACCA; *Il7*-Rev: GTTTGTGTGCCTTGTGATACTGTTAG.

### *In vitro* limiting dilution B cell generation assay

Experiments were performed as previously described (34). Briefly, OP9 stromal cells were plated on flat-bottom 96-well plates 1 day before the initiation of co-cultures, at a concentration of 3,000 cells per well. The following day stromal cells were γ-irradiated (3000 rad) and the sorted EPLM cells were added at different concentrations. Cultures were maintained in IMDM medium supplemented with 5 × 10^−5^ M β-mercaptoethanol, 1 mM glutamine, 0.03% (wt/vol) primatone, 100 U/mL penicillin, 100 μg/mL streptomycin, 5% FBS and 10% IL-7-conditioned medium. After 10 days in culture all wells were inspected under an inverted microscope and wells containing colonies of more than 50 cells were scored as positive.

### *In vivo* hematopoietic reconstitution assays

Ten million BM or LN cells from FLtgxIL7tg mice were injected intravenously into CD45.1^+^ recipient mice, which had been sub-lethally irradiated (400 rad) ~2 h before injection. Mice were euthanized 12–16 weeks after cell transfer and their spleen, thymus and bone marrow was analyzed for the presence of donor cells. For secondary transplantations, 6 × 10^6^ BM cells from recipient mice were injected intravenously into sub-lethally irradiated CD45.1^+^ recipients, in the same way. Secondary recipient spleens were analyzed after 9 weeks. For assessment of the *in vivo* B cell potential of EPLM, 6 × 10^4^ Ly6D^+^ EPLM sorted from the BM or LN of FLtgxIL7tg mice were intravenously injected into NOD/SCID/*Il2r*γ^−/−^ lymphopenic mice. Recipient spleens were analyzed for the presence of CD19^+^IgM^+^ cells 3 weeks after cell transfer.

### Statistical analysis

One-way ANOVA followed by a Tukey-test to correct for multiple comparisons between mouse genotypes was used. Statistical significance is indicated with asterisks in graphs. Non-significant differences are not indicated in the figures.

## Results

### Expansion of hematopoietic cells in secondary lymphoid organs of FLtgxIL7tg mice

**Figure 1:**
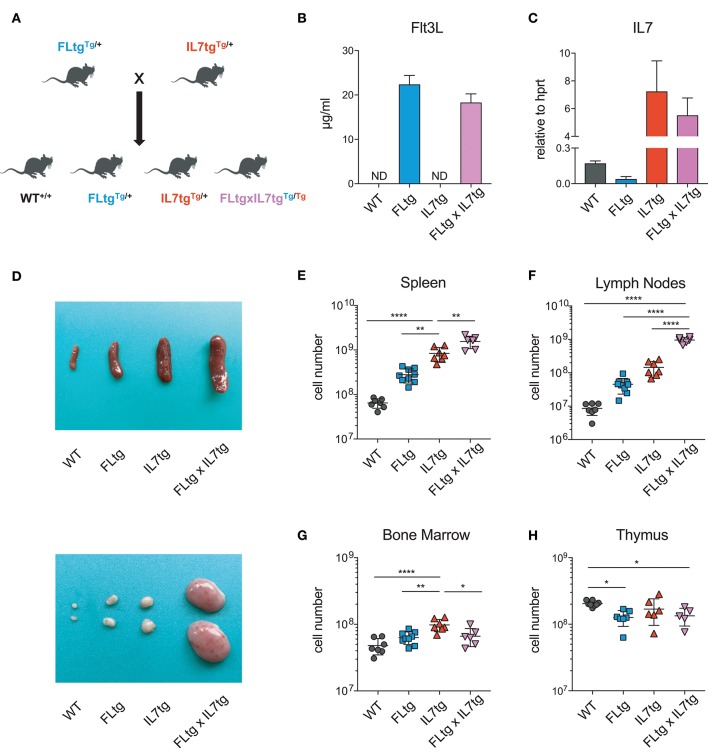
Increased cellularity of FLtgxIL7tg lymphoid organs. **(A)** Scheme of the breeding applied to obtain FLtgxIL7tg mice. **(B)** ELISA for human FL protein quantification in the serum of WT, FLtg, IL7tg, and FLtgxIL7tg mice (*n* = 4). **(C)** Quantitative PCR for the detection of *Il7* mRNA in the spleen of WT, FLtg, IL7tg, and FLtgxIL7tg mice (*n* = 3). Bars in **(B,C)** represent mean ± standard deviation. **(D)** Representative spleens (top) and lymph nodes (bottom) of WT, FLtg, IL7tg and FLtgxIL7tg mice. **(E–H)** Total numbers of live, nucleated cells in the spleen **(E)**, lymph nodes (axillary and inguinal) **(F)**, bone marrow (femurs and tibias) **(G)**, and thymus **(H)** of WT, FLtg, IL7tg, and FLtgxIL7tg mice. ^*^p < 0.05, ^**^p < 0.01, and ^****^p < 0.0001. Error bars indicate standard deviation.

FLtg and IL7tg mice heterozygous for the corresponding transgenes were crossed, resulting in mice carrying both transgenes (FLtgxIL7tg), as well as wild-type (WT) and single-transgenic littermates, which were used as controls (Figure 1A). FLtgxIL7tg mice were viable but at around 5–6 weeks after birth, they developed large, clearly visible inguinal lymph nodes (LN), which continued to grow and therefore mice had to be euthanized at the age of 9–10 weeks. None of the littermate controls exhibited this phenotype. All analyses of FLtgxIL7tg mice and their WT and single transgenic counterparts presented herein were done in 6–9 week old mice. We detected a massive increase in the amount of FL in the serum of FLtg and FLtgxIL7tg mice, which reached 22 and 18 ug/ml, respectively (Figure 1B). Even though IL-7 could not be detected in the serum of FLtgxIL7tg mice, as shown previously by *in situ* hybridization in IL7tg mice (35), a significant increase in *Il7* mRNA transcripts was observed in spleens of both IL7tg and FLtgxIL7tg mice (Figure 1C). Macroscopically, double transgenic mice exhibited a profound splenomegaly, with spleen size and average cellularity significantly larger than in single transgenic mice, in which the spleen was already increased compared to WT (Figures 1D,E). LN enlargement was even more striking, as shown in Figure 1D, with the average number of nucleated cells in all four inguinal and axillary LN reaching almost 10^9^ cells, compared to 3.4 × 10^6^ for WT, 45.4 × 10^6^ for FLtg and 145 × 10^6^ for IL7tg mice (Figure 1F). All other LN examined macroscopically (brachial, mediastinal) showed similar enlargement compared to WT and single transgenic mice. FLtgxIL7tg BM cellularity was somewhat increased compared to WT (less than 2-fold and not statistically significant) and similar to the single transgenic controls (Figure 1G). On the contrary, thymus cellularity was slightly decreased in single and double transgenic mice compared to their WT littermates (Figure 1H).

Analysis of the enlarged spleens and LN of FLtgxIL7tg mice showed that several mature hematopoietic cells, which normally reside in these secondary lymphoid tissues, were remarkably increased. Thus, spleen and LN Gr1^+^CD11b^+^ cells, including neutrophils and macrophages, were clearly increased in response to elevated FL levels (Supplementary Figure 1A). Also, and in agreement with what has been described previously in single FLtg mice (32), DC populations, including conventional DC of both types (CD11c^+^MHC-II^+^XCR1^+^ cDC1 and CD11c^+^MHC-II^+^XCR1^−^ cDC2), as well as B220^+^SiglecH^+^ plasmacytoid DC (pDC), were all dramatically increased in response to FL over-expression (Supplementary Figures 1B–E), although the statistical analysis did not show a significant effect on LN cDC1 and cDC2. A clear effect of over-expressing both cytokines was also observed on splenic and LN T cells. FLtgxIL7tg mice had increased numbers of both CD4^+^ and CD8^+^ T cells in spleen (4.4- and 13-fold increase compared to WT, respectively) and LN (11- and 30-fold increase compared to WT, respectively) (Supplementary Figures 2A,B). This effect was also seen in the numbers of the CD4^+^Foxp3^+^ regulatory T cell (Treg) fraction of CD4^+^ T cells, particularly in the LN (Supplementary Figures 2A,B), as shown before under conditions of high FL availability (36). This increase in peripheral T cell numbers was probably not due to increased thymic output, since analysis of T cell developmental stages in the thymi of FLtgxIL7tg mice showed that over-expression of both cytokines did not significantly affect the numbers of T cell progenitors, including CD4^+^ and CD8^+^ single positive and CD4/CD8 double positive pro-T cells. Interestingly, FL over-expression resulted in a reduction in the numbers of the earliest double negative T cell progenitors (Supplementary Figures 2C–E).

**Figure 2:**
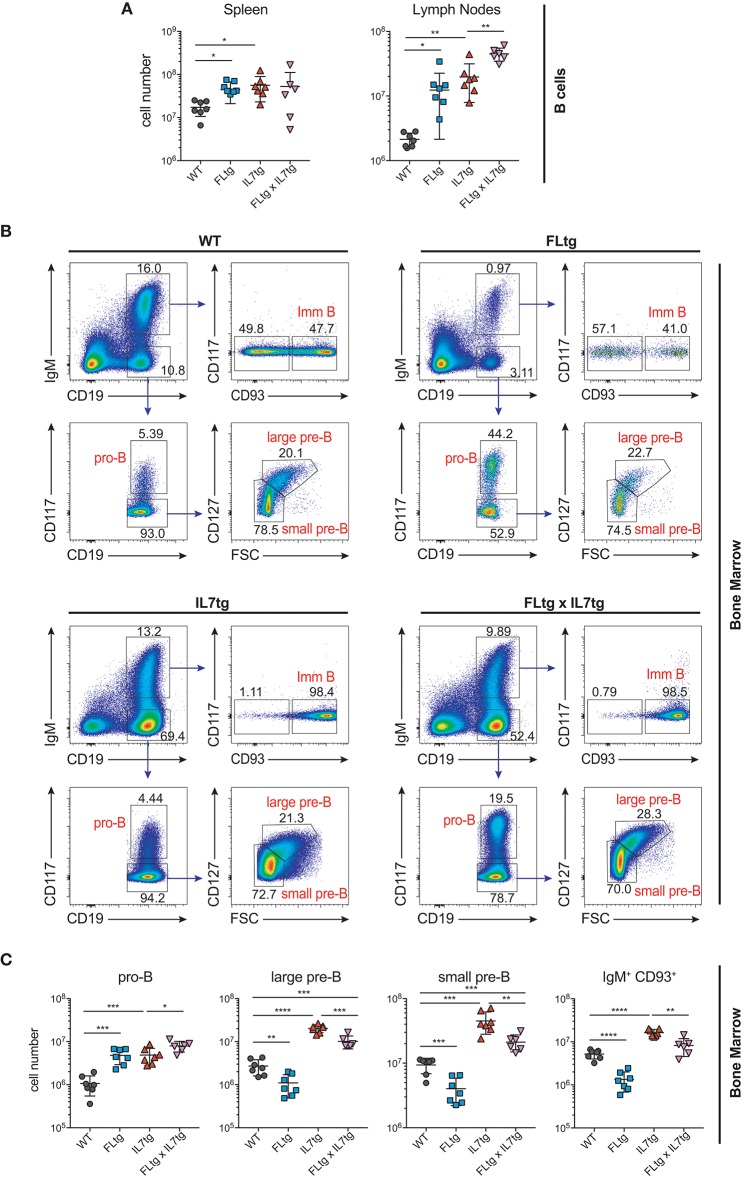
Expansion of B cell populations in lymphoid organs of FLtgxIL7tg mice. **(A)** Numbers of IgM^+^CD93^−^ mature recirculating B cells in the spleen (left) and lymph nodes (axillary and inguinal) (right) of WT, FLtg, IL7tg, and FLtgxIL7tg mice. **(B)** Representative FACS plots for the identification of B cell progenitor stages in the bone marrow of WT (upper left), FLtg (upper right), IL7tg (lower left), and FLtgxIL7tg (lower right) mice. Gate numbers indicate frequencies of parent gate. **(C)** Numbers of pro-B (CD19^+^IgM^−^CD117^+^), large pre-B (CD19^+^IgM^−^CD117^−^CD127^+^FSC^large^), small pre-B (CD19^+^IgM^−^CD117^−^CD127^−^FSC^small^) and immature B (CD19^+^IgM^+^CD93^+^) in the bone marrow of WT, FLtg, IL7tg, and FLtgxIL7tg mice. B cell populations were identified as indicated in B. ^*^p < 0.05, ^**^p < 0.01, ^***^p < 0.001, ^****^p < 0.0001. Error bars indicate standard deviation.

We next examined B cell populations in the peripheral lymphoid organs of FLtgxIL7tg and single transgenic mice. Analysis of peritoneal B cells showed that IL-7 over-expression significantly increased the percent of B2 cells, whereas the frequency of the IL-7-independent (37) CD19^+^CD5^+^CD11b^low^ B1a population was decreased by over-expression of both cytokines (Supplementary Figures 3A,B). Even though FL has been shown to be critical for the generation of B1a cells (30, 38), their relative frequency was not significantly increased upon FL over-expression, but rather slightly decreased, possibly due to the large expansion of myeloid cells in the peritoneal cavity (Supplementary Figure 3B). Mature, recirculating IgM^+^CD93^−^ B cells were significantly increased in the LN of FLtgxIL7tg mice compared to WT (21-fold), whereas over-expressing either cytokine alone resulted in increased mature B cell numbers compared to WT, but to a smaller extent (Figure 2A). Mature IgM^+^ B cells do not express CD135 and, unlike peripheral T cells, they are also CD127^−^ and hence do not respond to either FL or IL-7. We therefore reasoned that the observed increase in their numbers in the periphery was the result of increased B cell progenitor numbers in the BM, as shown previously in IL7tg mice (29). Analysis of BM B cell progenitor populations showed a moderate 1.6-fold increase in the numbers of IgM^+^CD93^+^ immature B cells, the population that exits the BM to recirculate in the periphery (Figures 2B,C). IgM^−^ B cell progenitors showed a more pronounced increase compared to WT controls: 7.4-fold for CD19^+^CD117^+^IgM^−^ pro-B cells, 3.7-fold for CD19^+^CD117^−^IgM^−^CD127^+^FSC^large^ large pre-B and 2.3-fold for CD19^+^CD117^−^IgM^−^CD127^−^FSC^small^ small pre-B cells (Figures 2B,C). This increase in BM CD19^+^ B cell progenitors was mainly an effect of elevated IL-7 levels, since FL over-expression seemed to have a negative outcome on the numbers of pre-B and immature B cells (~2-fold decreased in FLtgxIL7tg mice compared to IL7tg). Thus, the reduction in pre-B and immature B cell numbers observed in FLtg mice (32) is also present when IL-7 is abundantly available (in FLtgxIL7tg mice) and is therefore unlikely to be caused by decreased IL-7 availability, as previously hypothesized.

### Accumulation of lymphoid progenitors in peripheral lymphoid organs of FLtgxIL7tg mice

**Figure 3:**
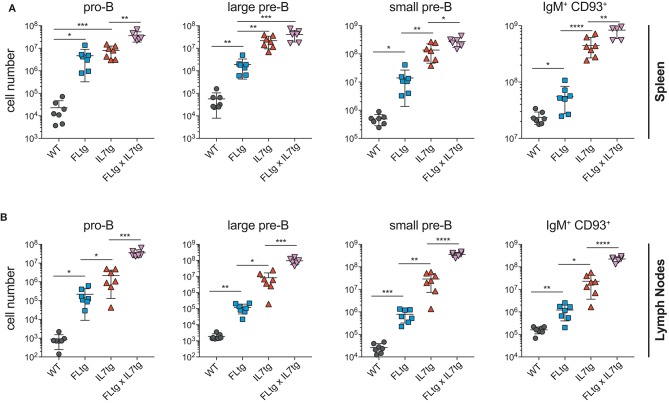
Accumulation of B cell progenitors in the spleen and lymph nodes of FLtgxIL7tg mice. Numbers of pro-B (CD19^+^IgM^−^CD117^+^), large pre-B (CD19^+^IgM^−^CD117^−^CD127^+^FSC^large^), small pre-B (CD19^+^IgM^−^CD117^−^CD127^−^FSC^small^) and immature B (CD19^+^IgM^+^CD93^+^) in the spleen **(A)** and lymph nodes (axillary and inguinal) **(B)** of WT, FLtg, IL7tg and FLtgxIL7tg mice. B cell progenitors were identified by FACS as shown in Figure 2B. ^*^p < 0.05, ^**^p < 0.01, ^***^p < 0.001, and ^****^p < 0.0001. Error bars indicate standard deviation.

Previous analysis of IL7tg mice has shown an abundance of immature B cell progenitors in the spleen and LN, where these populations are normally not found in significant numbers (29). We therefore analyzed spleen and LN of FLtgxIL7tg mice for the presence of early B cell progenitors. Indeed, in both spleen and LN of IL7tg mice, all stages of CD19^+^ committed B cell progenitors were detected (Figures 3A,B). Contrary to what was observed in the BM, however, additional FL over-expression, in FLtgxIL7tg mice, further increased the numbers of pro-B (4.6-fold in spleen and 16-fold in LN), large pre-B (1.8-fold in spleen and 11-fold in LN), small pre-B (2-fold in spleen and 12-fold in LN) and immature B (1.9-fold in spleen and 9.8-fold in LN) cells. Thus, FL and IL-7 seem to act synergistically in promoting the accumulation of immature B cell progenitors in the peripheral lymphoid organs of mice over-expressing both cytokines.

**Figure 4:**
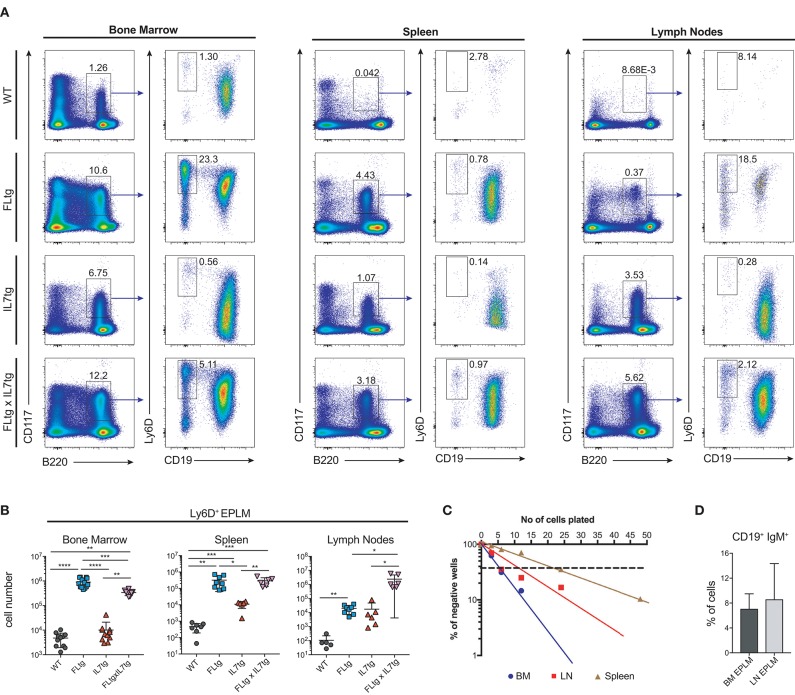
Ly6D^+^ EPLM accumulate in the spleen and lymph nodes of FLtgxIL7tg mice. **(A)** Representative FACS plots for the identification of Ly6D^+^ EPLM in the bone marrow (femurs and tibias) (Left), spleen (Middle) and lymph nodes (axillary and inguinal) (Right) of WT, FLtg, IL7tg, and FLtgxIL7tg mice. Gate numbers indicate frequencies of parent gate. In every panel the initial plot (CD117 vs. B220) shows live, SiglecH^−^CD11c^−^NK1.1^−^ cells. **(B)** Numbers of Ly6D^+^ EPLM (NK1.1^−^CD11c^−^SiglecH^−^CD19^−^B220^int^CD117^int^Ly6D^+^) in the bone marrow, spleen and lymph nodes of WT, FLtg, IL7tg and FLtgxIL7tg mice. **(C)** *In vitro* differentiation potential of Ly6D^+^ EPLM from bone marrow (blue), spleen (red) and lymph nodes (brown) of FLtgxIL7tg mice. Cells were plated on OP9 stromal cells + IL-7 at different cell densities and the number of wells containing B cells were scored under the microscope after 10 days. One representative of three independent experiments is shown. **(D)** *In vivo* B cell reconstitution potential of Ly6D^+^ EPLM from bone marrow (black) or lymph nodes (gray) of FLtgxIL7tg mice. Six thousand Ly6D^+^ EPLM were intravenously injected into NOD/SCID/*Il2r*γ^−/−^ mice (*n* = 5) and the percentage of CD19^+^IgM^+^ donor cells was evaluated in the spleen of recipient animals 3 weeks after transplantation. ^*^p < 0.05, ^**^p < 0.01, ^****^p < 0.0001. Error bars indicate standard deviation.

Since CD135 is not detectable on CD19^+^ B cell progenitors, this additional rise in their numbers in spleen and LN upon simultaneous FL and IL-7 over-expression is unlikely to be a direct effect of FL on their survival and/or proliferation. However, their immediate precursors, Ly6D^+^ EPLM, are CD135^+^ and have been shown to increase dramatically in response to elevated FL levels (13, 34, 39). Therefore, we assessed their numbers in the BM and periphery of FLtgxIL7tg mice. As shown in Figures 4A,B, CD11c^−^NK1.1^−^SiglecH^−^CD19^−^B220^int^CD117^int^Ly6D^+^ EPLM were indeed dramatically increased upon FL over-expression in the BM, whereas IL-7 over-expression did not increase their numbers and even reduced them when in combination with high FL expression (Figure 4B, FLtg compared to FLtgxIL7tg). Ly6D^+^ EPLM were clearly detected in spleens of FLtg and FLtgxIL7tg mice, whereas in LN they were strikingly expanded mainly upon over-expression of both cytokines. FLtg and IL7tg LN also had higher numbers of Ly6D^+^ EPLM compared to WT, in which they were barely detectable. However, this increase was not statistically significant and was proportional to the corresponding overall increase in LN total cellularity (Figure 1F), whereas FLtgxIL7tg LN Ly6D^+^ EPLM were 120- and 137-fold increased compared to their FLtg and IL7tg counterparts, respectively. This expansion is ~10 times higher than the corresponding LN cellularity difference between these mouse genotypes, indicating that FL and IL-7 synergistically promote the accumulation and/or expansion of Ly6D^+^ EPLM in the LN of FLtgxIL7tg mice. Since Ly6D^+^ EPLM are not yet committed to the B cell lineage (39, 40), we assessed the ability of these spleen- and LN-residing FLtgxIL7tg progenitors to generate B cells *in vitro* and *in vivo*. Sorting and plating FLtgxIL7tg Ly6D^+^ EPLM on OP9 stromal cells together with IL-7 under limiting dilution conditions showed that these cells were able to give rise to CD19^+^ B cells *in vitro* (Figure 4C). We noticed that the frequency of LN-derived Ly6D^+^ EPLM that could generate B cells under these conditions was slightly reduced compared to their BM counterparts, which showed a frequency similar to WT BM-derived Ly6D^+^ EPLM (13, 39). However, after transplantation into NOD/SCID/*Il2r*γ^−/−^ lymphopenic mice, LN-derived FLtgxIL7tg Ly6D^+^ EPLM were as capable as their BM-derived counterparts at generating IgM^+^ B cells *in vivo* (Figure 4D). Thus, under conditions of simultaneous increase in FL and IL-7 availability, functional CD19^−^Ly6D^+^ EPLM progenitors can reside and accumulate in the spleens and LN of mice.

### Hematopoietic progenitors with long-term, multilineage reconstitution capacity reside in the LN of FLtgxIL7tg mice

**Figure 5:**
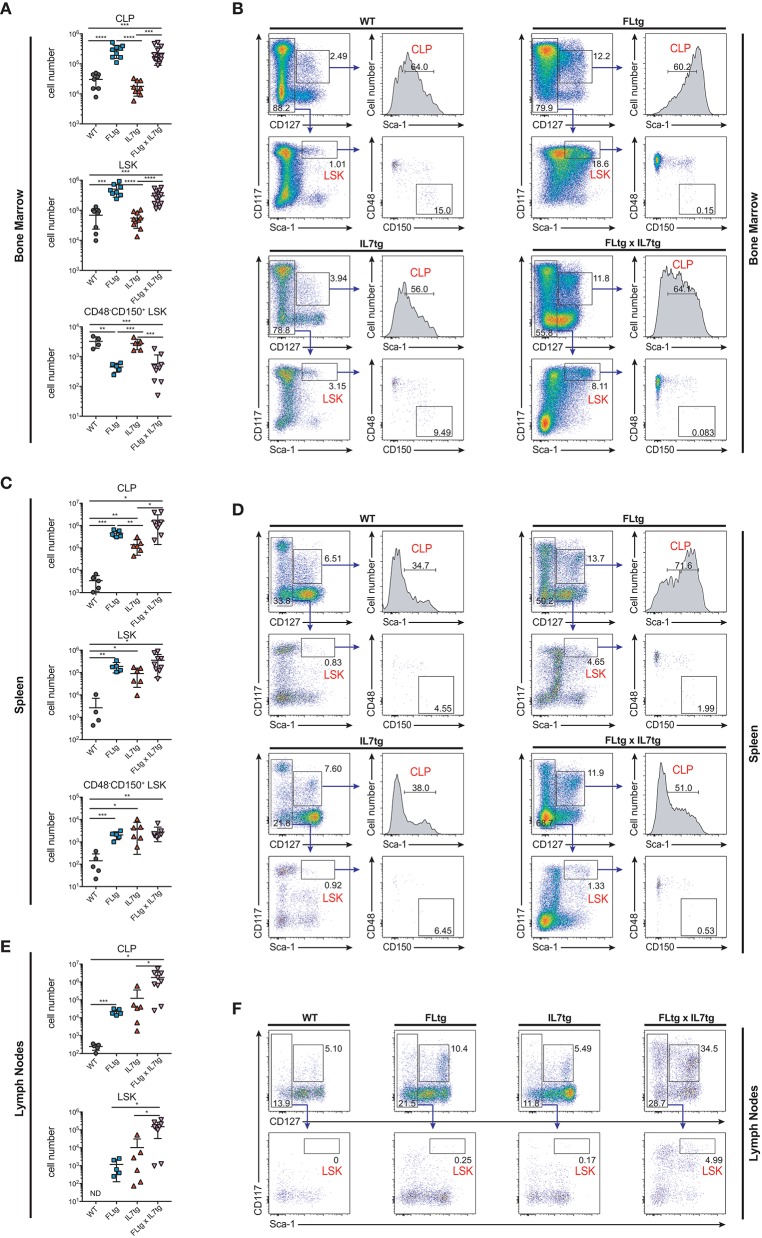
Increased numbers of CLP and LSK in the spleen and lymph nodes of FLtgxIL7tg mice. **(A)** Numbers of Lin^−^CD117^int^Sca1^int^CD127^+^ (CLP), Lin^−^CD127^−^CD117^+^Sca1^+^ (LSK) and Lin^−^CD127^−^CD117^+^Sca1^+^CD48^−^CD150^+^ LSK in the bone marrow of WT, FLtg, IL7tg, and FLtgxIL7tg mice. **(B)** Representative FACS plots for the identification of CLP, LSK, and CD48^−^CD150^+^ LSK in the bone marrow (femurs and tibias) of WT, FLtg, IL7tg, and FLtgxIL7tg mice. Gate numbers indicate frequencies of parent gate. In every panel the upper left plot (CD117 vs. CD127) shows live, Lineage^−^ cells. **(C)** Numbers of CLP, LSK and CD48^−^CD150^+^ LSK in the spleens of WT, FLtg, IL7tg, and FLtgxIL7tg mice. **(D)** Representative FACS plots for the identification of CLP, LSK, and CD48^−^CD150^+^ LSK in the spleens of WT, FLtg, IL7tg, and FLtgxIL7tg mice. Gate numbers indicate frequencies of parent gate. In every panel the upper plot (CD117 vs. CD127) shows live, Lin- cells. **(E)** Numbers of CLP and LSK in the LN of WT, FLtg, IL7tg, and FLtgxIL7tg mice. **(F)** Representative FACS plots for the identification of LSK in the LN of WT, FLtg, IL7tg, and FLtgxIL7tg mice. Gate numbers indicate frequencies of parent gate. In every panel the upper plot (CD117 vs. CD127) shows live, Lin- cells. *p < 0.05, **p < 0.01, ***p < 0.001, and ****p < 0.0001. Error bars indicate standard deviation.

Apart from Ly6D^+^ EPLM, CLP and LSK cells are greatly expanded in FLtg mice (32). We therefore investigated their potential expansion in the BM and presence in the spleen and LN of FLtgxIL7tg mice. Under conditions of high *in vivo* FL availability, CD135 becomes undetectable by flow cytometry (32), possibly due to the continuous binding of the ligand to its receptor. Thus, we identified CLP using the original Lin^−^CD117^int^Sca1^int^CD127^+^ phenotype (14) (Figure 5B). We found CLP numbers to be significantly increased in the BM of mice over-expressing FL, whereas IL-7 over-expression did not have any effect (Figure 5A). This was also the case in the spleen, where splenic FLtgxIL7tg CLP were increased compared to WT (Figure 5C). Similarly to EPLM, we observed a greater than 10-fold increase in LN FLtgxIL7tg CLP numbers compared to their single transgenic counterparts, which were already increased compared to WT (Figures 5E,F). CD127^−^ LSK cell analysis showed a similar picture, with LSK being greatly increased in the BM and found in significant numbers in the spleen and LN of FLtgxIL7tg mice. Within the LSK compartment, the CD48^−^CD150^+^ phenotype can be used to enrich for hematopoietic stem cells (HSC) with multilineage developmental potential and self-renewal capacity (41). As shown in Figure 5A (bottom), CD48^−^CD150^+^ LSK cells were significantly reduced in the BM of FLtg mice and this was also the case in FLtgxIL7tg BM (6-fold decreased compared to WT). These cells were detected at very low frequency in the spleen of FLtgxIL7tg mice (Figure 5C), and we could not detect them in the LN of any of the mouse genotypes analyzed (data not shown).

**Figure 6:**
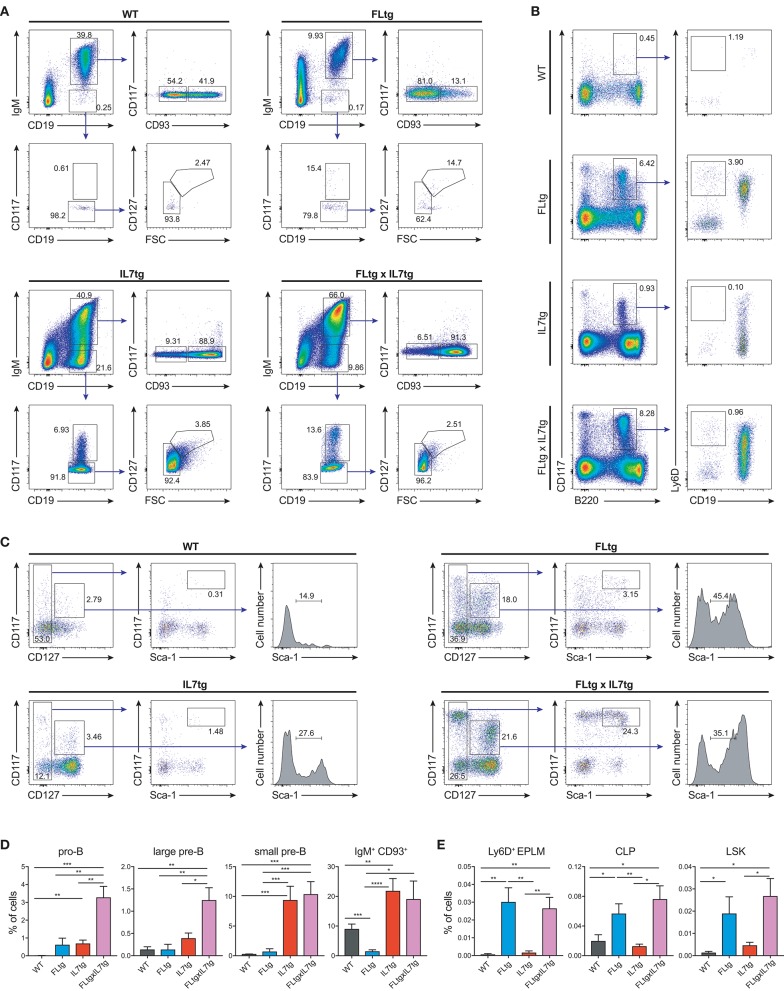
Hematopoietic progenitors in the blood of FLtgxIL7tg mice. **(A)** Representative FACS plots for the identification of B cell progenitor stages in the blood of WT (upper left), FLtg (upper right), IL7tg (lower left) and FLtgxIL7tg (lower right) mice. Gate numbers indicate frequencies of parent gate. **(B)** Representative FACS plots for the identification of Ly6D^+^ EPLM in the blood of WT (upper left), FLtg (upper right), IL7tg (lower left), and FLtgxIL7tg (lower right) mice. Gate numbers indicate frequencies of parent gate. In every panel the left plot (CD117 vs. B220) shows live, NK1.1^−^CD11c^−^SiglecH^−^ cells. **(C)** Representative FACS plots for the identification of LSK and CLP in the blood of WT (upper left), FLtg (upper right), IL7tg (lower left), and FLtgxIL7tg (lower right) mice. Gate numbers indicate frequencies of parent gate. In every panel the left plot (CD117 vs. CD127) shows live, Lin^−^ cells. **(D)** Percentages of pro-B (CD19^+^IgM^−^CD117^+^), large pre-B (CD19^+^IgM^−^CD117^−^CD127^+^FSC^large^), small pre-B (CD19^+^IgM^−^CD117^−^CD127^−^FSC^small^) and immature B cell (CD19^+^IgM^+^CD93^+^) progenitors in the blood of WT (*n* = 9), FLtg (*n* = 11), IL7tg (*n* = 8), and FLtgxIL7tg (*n* = 11) mice. B cell progenitors were identified by FACS as shown in **(A)**. **(E)** Percentages of Ly6D^+^ EPLM (CD11c^−^NK1.1^−^SiglecH^−^B220^int^CD117^int^Ly6D^+^CD19^−^), CLP (Lin^−^CD117^int^Sca1^int^CD127^+^) and LSK (Lin^−^CD117^+^Sca1^+^CD127^−^) progenitors in the blood of WT (*n* = 6), FLtg (*n* = 6), IL7tg (*n* = 7), and FLtgxIL7tg (*n* = 10) mice. The y-axis on all plots indicates % of live, nucleated blood cells. *p < 0.05, **p < 0.01, ***p < 0.001, and ****p < 0.0001. Error bars indicate standard deviation.

Taken together, our analysis shows that over-expression of both FL and IL-7 leads to a dramatic increase in all BM CD135^+^ and CD127^+^ progenitors and to their migration to the periphery, presumably due to space constrains in the BM. In support of this hypothesis, all CD19^+^ B cell progenitor stages were found significantly increased in the peripheral blood of FLtgxIL7tg mice (Figures 6A,D). Strikingly, the same was true for CD135^+^ Ly6D^+^ EPLM, CLP and LSK progenitors, which were increased in the blood mainly in response to FL over-expression (Figures 6B,C,E). This effect of FL and IL-7 simultaneous over-expression in promoting the migration of cells from the BM to the periphery was seen mainly on progenitors, since mature B and T cell frequencies in the blood of FLtgxIL7tg mice were not dramatically changed, whereas the relative frequencies of cDC and NK cells were significantly increased in the blood of FLtg animals (Supplementary Figure 4). Thus, the synergistic effect of FL and IL-7 over-expression in expanding BM hematopoietic progenitors leads to their exit from the BM and accumulation not only in the spleen but also in LN, where some of these progenitors are undetectable in WT mice.

**Figure 7:**
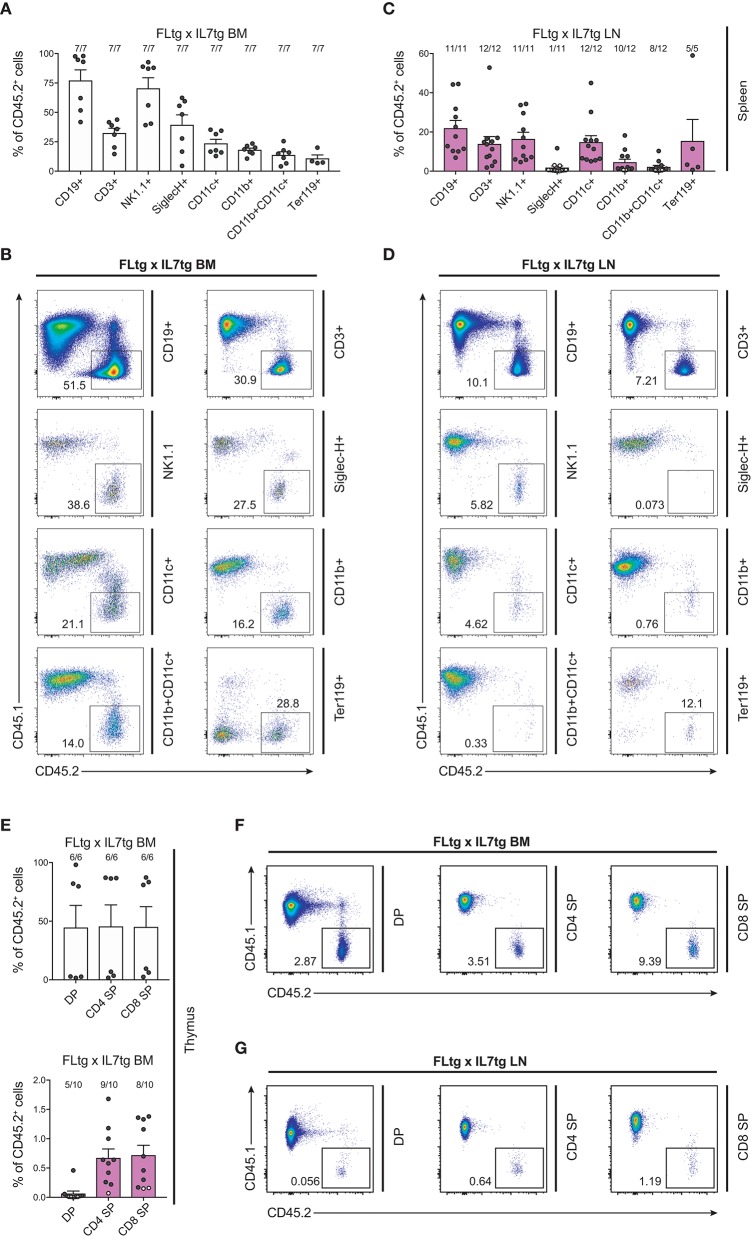
Hematopoietic reconstitution potential of FLtgxIL7tg LN cells. **(A,C)** Percentage of CD45.2^+^ donor cells within the indicated lineages in spleens of mice reconstituted with FLtgxIL7tg BM **(A)** or FLtgxIL7tg LN **(C)**. Results from four independently performed experiments are shown. Bars indicate mean ± standard error of the mean. Black circles represent mice where the corresponding lineage was scored as positive for the presence of donor-derived cells (>50 cells in the CD45.2^+^ gate) and white circles mice with no reconstitution (<50 cells in the CD45.2^+^ gate). The fraction of positive-to-total mice analyzed for each lineage is indicated above the corresponding bar. Lineages were defined as follows: CD19^+^: CD11b^−^CD11c^−^CD3^−^CD19^+^; CD3^+^: CD11b^−^CD11c^−^CD19^−^CD3^+^; NK1.1^+^: CD3^−^CD11c^−^B220^−^SiglecH^−^NK1.1^+^; SiglecH^+^: CD11b^−^NK1.1^−^B220^+^SiglecH^+^; CD11c^+^: NK1.1^−^SiglecH^−^B220^−^CD11b^−^CD11c^+^; CD11b^+^: NK1.1^−^SiglecH^−^B220^−^CD11c^−^CD11b^+^; CD11b^+^CD11c^+^: NK1.1^−^SiglecH^−^B220^−^CD11b^+^CD11c^+^; Ter119^+^: Ter119^+^. **(B,D)** Representative FACS plots showing the CD45.2^+^ donor population identified within the lineages shown in **(A,C)**. Left: recipients transplanted with FLtgxIL7tg BM; Right: recipients transplanted with FLtgxIL7tg LN. **(E)** Percentage of CD45.2^+^ donor cells within the indicated T cell populations in thymi of mice reconstituted with FLtgxIL7tg BM (upper) and FLtgxIL7tg LN (lower). Results from three independently performed experiments are shown. For FLtgxIL7tg BM: *n* = 6; for FLtgxIL7tg LN: *n* = 10. Bars indicate mean ± standard error of the mean. Black circles represent mice where the corresponding lineage was scored as positive for the presence of donor-derived cells (>50 cells in the CD45.2^+^ gate) and white circles mice with no reconstitution (<50 cells in the CD45.2^+^ gate). The ratio of positive-to-total mice analyzed for each lineage is indicated above the corresponding bar. Cells were identified as follows: DP: CD3^+^CD4^+^CD8^+^; CD4 SP: CD3^+^CD8^−^CD4^+^; CD8 SP: CD3^+^CD4^−^CD8^+^. **(F,G)** Representative FACS plots showing the CD45.2^+^ donor population identified within the indicated thymic T cell populations. Upper: recipients transplanted with FLtgxIL7tg BM; lower: recipients transplanted with FLtgxIL7tg LN.

We next sought to evaluate whether these multipotent hematopoietic progenitors identified by flow cytometry in the LN of FLtgxIL7tg mice were functional, i.e., if they had the ability to generate multiple hematopoietic lineages upon transplantation. To this end, we infused 10 × 10^6^ unfractionated LN cells from CD45.2^+^ FLtgxIL7tg, into sub-lethally irradiated congenic CD45.1^+^ WT mice. FLtgxIL7tg BM cells were used as a positive control for the long-term reconstitution of hematopoietic lineages, since they contain functional hematopoietic progenitors. Recipient mice were analyzed 12–16 weeks after transplantation for the contribution of CD45.2^+^ donor cells to the different hematopoietic lineages. As expected, overall engraftment of donor cells in the BM, spleen and thymus of recipient mice was significantly lower in LN-transplanted recipients compared to BM transplanted ones (Supplementary Figures 5A,B). Thus, in the FLtgxIL7tg LN transplanted hosts, 20% splenic, 5.4% BM and 6.8% thymic cells were of donor origin. Analysis of the spleen of FLtgxIL7tg LN-reconstituted mice showed that 21.8% CD19^+^ B cells, 13.8% CD3^+^ T cells, 16.3% NK1.1^+^ NK cells, 14.7% SiglecH^−^CD11b^−^CD11c^+^ DC, 4.5% CD11b^+^CD11c^−^ myeloid cells and 15% Ter119^+^ erythroid cells were of donor origin, with more than 80% of the transplanted mice showing donor-derived reconstitution in all the above lineages (Figures 7A–D). Donor contribution was also evaluated in the thymus of recipient mice, where we found small but clearly detectable populations of FLtgxIL7tg LN-derived CD4^+^, CD8^+^, and CD4/CD8 double positive T cell progenitors (Figures 7E–G). Furthermore, BM analysis showed that a significant fraction of CD19^+^ B cell progenitors were of FLtgxIL7tg LN donor origin, with the average percent ranging from 5.6% for large pre-B to 13% for immature IgM^+^CD93^+^ B cells (Supplementary Figures 5C,D). Remarkably, we also found small but clearly detectable populations of donor-derived CLP (5%) and LSK (3.5%) 12 weeks after transplantation. Thus, FLtgxIL7tg LN contain progenitors with the long-term capacity to generate multiple hematopoietic lineages.

**Figure 8:**
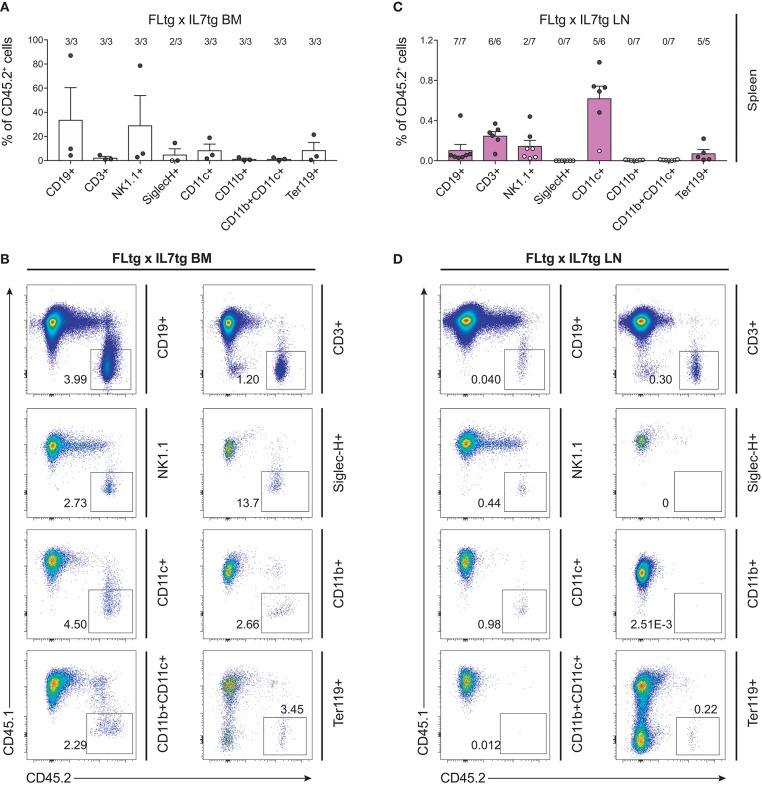
Hematopoietic reconstitution potential of FLtgxIL7tg LN cells after secondary transplantation. **(A,C)** Percentage of CD45.2^+^ donor cells within the indicated lineages in spleens of secondary CD45.1^+^ recipients, which received BM of CD45.1^+^ mice reconstituted with FLtgxIL7tg BM **(A) o**r FLtgxIL7tg LN **(C)**. Results from three independently performed experiments are shown. Bars indicate mean ± standard error of the mean. Black circles represent mice where the corresponding lineage was scored as positive for the presence of donor-derived cells (>50 cells in the CD45.2^+^ gate) and white circles mice with no reconstitution (<50 cells in the CD45.2^+^ gate). The ratio of positive-to-total mice analyzed for each lineage is indicated above the corresponding bar. Lineages were defined as follows: CD19^+^: CD11b^−^CD11c^−^CD3^−^CD19^+^; CD3^+^: CD11b^−^CD11c^−^CD19^−^CD3^+^; NK1.1^+^: CD3^−^CD11c^−^B220^−^SiglecH^−^NK1.1^+^; SiglecH^+^: CD11b^−^NK1.1^−^B220^+^SiglecH^+^; CD11c^+^: NK1.1^−^SiglecH^−^B220^−^CD11b^−^CD11c^+^; CD11b^+^: NK1.1^−^SiglecH^−^B220^−^CD11c^−^CD11b^+^; CD11b^+^CD11c^+^: NK1.1^−^SiglecH^−^B220^−^CD11b^+^CD11c^+^; Ter119^+^: Ter119^+^. **(B,D)** Representative FACS plots showing the CD45.2^+^ donor population identified within the lineages shown in **(A,C)**. Left: FLtgxIL7tg BM as primary donor; right : FLtgxIL7tg LN as primary donor.

The presence of early hematopoietic progenitors in the host BM 12 weeks after transfer of FLtgxIL7tg LN cells, prompted us to assess their self-renewal capacity by re-transplanting them into secondary recipients. Six million unfractionated BM cells from FLtgxIL7tg LN-reconstituted mice and FLtgxIL7tg BM-reconstituted controls were intravenously injected into congenic CD45.1^+^ irradiated WT hosts 12 weeks after the first transplantation. Nine weeks after secondary transfer, we could detect FLtgxIL7tg-derived donor cells in the secondary recipients' spleen. Importantly, CD45.2^+^ cells were found in multiple hematopoietic lineages. Thus, FLtgxIL7tg LN donor-derived cells were found in: 0.1% CD19^+^ B cells, 0.25% CD3^+^ T cells, 0.6% CD11c^+^ DC and 0.07% Ter119^+^ erythroid cells (Figures 8A–D). These results indicate that there are FLtgxIL7tg LN-residing multipotent hematopoietic progenitors with self-renewal capacity. Overall, considering the significant multilineage contribution of FLtgxIL7tg LN-derived donor cells in host hematopoietic reconstitution more than 12 weeks after transfer, as well as their presence in secondary transplanted hosts after another 9 weeks, we conclude that the LN of FLtgxIL7tg mice contain hematopoietic progenitors with *in vivo* long-term multi-lineage reconstitution ability, some of which have self-renewal capacity.

## Discussion

We have previously generated and analyzed mice with increased, sustained *in vivo* levels of either IL-7 (28, 29) or FL (13, 32, 39), which provided important insights to the roles of the two cytokines at different stages of the various lineages of hematopoiesis. In the present study we over-expressed both cytokines in order to evaluate the concerted action of both FL and IL-7 on the regulation of hematopoiesis in an *in vivo* setting. We find a synergistic effect of the two cytokines in promoting the generation and expansion of lymphoid cells, resulting in a profound enlargement of secondary lymphoid organs, such as spleen and LN, significantly more than what can be seen in single transgenic mice (Figure 1). Both spleen and LN of FLtgxIL7tg mice were populated by significant numbers of multipotent hematopoietic progenitors, which in WT mice are generally confined to the BM.

The enlarged spleens and LN of FLtgxIL7tg mice contained significantly increased populations of myeloid cells (Supplementary Figure 1A). This probably reflected the expansion in BM myeloid progenitors that FL over-expression induces (32). Indeed, when assessing early myelo-erythroid progenitor stages in FLtgxIL7tg BM, we observed a significant increase in the earliest identified myeloid progenitors, pre-GM and GMP (42), whereas early erythroid/megakaryocyte progenitors were decreased (Supplementary Figure 6), in accordance with the anemia and thrombocytopenia caused by elevated FL (32). Generation of DC is known to depend on FL and we indeed found all splenic and some LN DC populations significantly increased (Supplementary Figure 1). This could be the result of DC expansion in peripheral lymphoid organs, since these cells are CD135^+^, or could be due to FL-mediated expansion of their progenitors. Due to the inability to stain for CD135^+^ in mice over-expressing FL (32) we were not able to assess the numbers of cDC and pDC progenitors (43–45).

Peripheral T cells were also increased dramatically in spleens and LN of FLtgxIL7tg mice, even though thymic size and T cell output was not increased. Thus, this seems to be mainly an effect of IL-7, which is known to regulate homeostasis of peripheral T cells, particularly of CD8^+^ (46) which was the T cell population with the biggest expansion in FLtgxIL7tg mice (Supplementary Figures 2A,B). Interestingly, FL over-expression alone also resulted in some increase in mature T cell numbers. Since these cells are CD135^−^, we postulate that this is an indirect effect of high FL levels. Previous experiments, showing expansion of regulatory T cells upon increased FL availability, suggested that this is mediated by IL-2 produced by the expanded DC (36), thus providing a potential explanation for the observed increase in peripheral T cells when FL is over-expressed. The somewhat reduced thymopoiesis observed in FLtgxIL7tg mice might be a direct or secondary result of high FL expression by thymic stromal cells, since IL-7 over-expression alone in the IL7tg mouse model used herein did not affect T cell development (35). Since thymus seeding progenitors that migrate from the BM are multipotent and express CD135 (47, 48), it is conceivable that under the influence of increased FL levels a larger fraction of them differentiates toward myeloid or DC fates, thus resulting in somewhat reduced CD4/CD8 double-negative numbers.

The synergistic effect of FL and IL-7 is clearly manifested in the generation of B cells. Expression of the receptors for the two cytokines on B cell generating progenitors occurs at slightly different developmental stages: CD135 is expressed on early progenitors (LSK, CLP, EPLM) and is down-regulated upon Pax5/CD19 expression and commitment to the B cell lineage, whereas CD127 is expressed from the CLP up to the small pre-B stage. Accordingly, both receptors are co-expressed during the CLP/EPLM developmental stage, in which a potential synergy between the two cytokines in promoting B cell development can occur. FL is mainly acting as a proliferative factor for CLP/EPLM progenitors, whereas IL-7 supports their survival, rather than expanding them (13). Thus, we find the number of these progenitors greatly expanded in the BM of FLtg and FLtgxIL7tg mice, while IL-7 over-expression does not further increase their numbers but rather decreases them slightly (Figures 4B, 5A). IL-7 acts as a proliferative factor for CD19^+^ B cell progenitors in the BM, leading to a 5- to 9-fold increase in their numbers upon over-expression (when comparing IL7tg to WT or FLtgxIL7tg to FLtg). This effect of the two cytokines on the proliferation of hematopoietic progenitors was confirmed in the present study, since we found a significant increase in the percentage of cycling LSK in the BM of mice over-expressing FL, whereas IL-7 had a proliferative effect mainly on CD19^+^ B cell progenitors (Supplementary Figure 7). This vast expansion of CD19^+^ cells upon IL-7 over-expression could explain the slight reduction in FLtgxIL7tg Ly6D^+^ EPLM numbers compared to FLtg mice, since the expanded progenitor populations have to compete for limited space in the BM. By contrast, in peripheral lymphoid tissues, such as the spleen and LN, which can enlarge to accommodate more cells, the synergistic effect of FL and IL-7 can be clearly seen, as FLtgxIL7tg spleen and LN contain significantly more CLP/EPLM and CD19^+^ B cell progenitors of all stages compared to their single transgenic counterparts. Interestingly, the proliferative effect of the two cytokines observed in the BM was also seen in the LN (Supplementary Figure 7), indicating that progenitors continue to expand in response to the two cytokines after their migration to the periphery. The space restrictions imposed on cells in the BM might also be the reason for the observed reduction in the number of BM pre-B and immature cells when FL is over-expressed. We have previously hypothesized that this might be the result of reduced IL-7 availability in the BM of FLtg mice, but as the same effect seems to occur in FLtgxIL7tg mice, this is unlikely to be the reason. It is still possible that other trophic signals required for the expansion and/or survival of pre-B cells become limiting in the BM when CD135^+^ progenitors expand massively, as is the case in FLtg and FLtgxIL7tg mice. However, this does not happen in the periphery, where the FL-mediated expansion of CLP/EPLM results in a corresponding increase in pro-B and pre-B cell numbers. Taken together, FL and IL-7 act in concert to promote B cell development, FL by providing sufficient numbers of CLP/EPLM progenitors and IL-7 by promoting their survival and further expansion after commitment to the B cell fate.

FL over-expression resulted in a major expansion of LSK cells, which are largely CD135^+^. When IL-7 was additionally over-expressed, this resulted in the detection of significant LSK numbers in the spleen and LN of FLtgxIL7tg mice. Since LSK are CD127^−^, we hypothesize that the reason for their increase mainly in FLtgxIL7tg LN is again related to confined space and/or resources in the FLtgxIL7tg BM, thus leading to their migration to peripheral lymphoid organs when expanded by FL over-expression. In support of this hypothesis, LSK can also be detected in the blood of FLtgxIL7tg mice (Figures 6C,E). Expression of molecules associated with progenitor migration from the BM, such as S1PR_1_, CD44, and CXCR4 was not dramatically different between genotypes, with the exception of an FL-mediated increase in the CXCR4^+^ fraction of B cell progenitors, which might be an indirect effect of FL, as these cells are CD135^−^ (Supplementary Figure 8). This indicates that it is mainly competition for BM space/resourses that leads to their accumulation in peripheral lymphoid organs. LSK are mostly comprised of multipotent progenitors with mixed lineage potentials and biases, but which are not considered to possess self-renewal capacity (7). Self-renewing HSC within the LSK compartment can be enriched for by staining with the SLAM markers CD48 and CD150, and are contained within the CD48^−^CD150^+^ LSK fraction (41). We were not able to detect CD48^−^CD150^+^ LSK cells in the LN of FLtgxIL7tg mice. However, as reported previously (32), and seen in Figure 5A, the CD48^−^CD150^+^ fraction of LSK in the BM is severely reduced in numbers upon FL over-expression. These cells are CD135^−^ when identified by flow cytometry, although some of them express mRNA for the receptor (49). Therefore we do not know if this reduction is a direct effect of FL-signaling. In addition, we cannot exclude that high FL availability might affect the expression of SLAM markers, thus resulting in some HSC losing the CD48^−^CD150^+^ phenotype in FLtg and FLtgxIL7tg mice. The expression level of other important markers for the identification of progenitor stages, such as CD117 and Sca1, might also be changed upon FL over-expression. As shown in Figures 5B,D, Sca1 expression in the majority of FLtg and FLtgxIL7tg CLP is relatively higher than the one of their WT and IL7tg counterparts. The same is true for CD117 expression in pro-B (Figure 2B). This might be the result of altered marker expression or a selective expansion of the (few in WT mice) cells expressing high levels of the corresponding proteins.

In order to functionally assess the precursor activity of hematopoietic progenitors found in the LN of FLtgxIL7tg mice, we transplanted LN cells from these mice into myelo-ablated WT recipients and assessed their long-term multilineage reconstitution capacity. We found a significant contribution of FLtgxIL7tg donor cells in lymphoid, myeloid, dendritic and erythroid lineages 12–16 weeks after transplantation. This is indicative of the presence of multipotent progenitors in the double transgenic LN, since myeloid and dendritic cells are not long-lived and therefore these donor cells could not be mature myeloid/dendritic cells transferred from the FLtgxIL7tg LN. In addition, we found donor contribution in all stages of recipient T cell development, suggesting that the FLtgxIL7tg LN contain progenitors with thymus-seeding potential. The exact nature of these precursors is not known, but they are known to have multilineage developmental capacity (47, 48). Furthermore, we detected donor progenitor cells in the BM of recipient animals, which upon transplantation into secondary recipients showed a small but clearly detectable contribution in the regeneration of different lineages. We conclude from this data that the LN of FLtgxIL7tg mice contain hematopoietic progenitors with long-term multilineage hematopoietic regeneration capacity. These could be HSC, which do not display the CD48^−^CD150^+^ phenotype due to alterations in SLAM marker expression, or downstream multipotent progenitors that have acquired self-renewal capacity under conditions of high FL availability – possibly by an autocrine FL effect.

Irrespective of the precise nature of the multipotent hematopoietic progenitor that resides in FLtgxIL7tg LN, it is clear from our data that not only the spleen but also the LN of these double transgenic mice can support the survival of immature precursors, such as LSK, CLP, EPLM, and pre-B cells, which are normally only found in the BM. Extra-medullary hematopoiesis has been described in patients and has been mostly associated either with bone marrow failure or with myeloproliferative disease (50, 51). Similarly, disruption of hematopoiesis in mice caused by drug treatment (52), mutations (53–55) or cytokine over-expression (56, 57) can lead to extra-medullary hematopoiesis. However, the main extra-medullary site where hematopoietic progenitors are detected in both patients and mice is the spleen, whereas no such precursors have been reported in LN in these cases. Hematopoietic progenitors have been shown to circulate to the periphery through blood and lymph, but only very small numbers have been detected in lymph under normal conditions and they were practically undetected in LN (58). A human NK precursor has been detected in LN (59), while it has been shown that at early time points after BM transplantation T cell lymphopoiesis can occur in extra-thymic sites, including LN (60). Interestingly, repeated administration of FL in mice has led to pronounced presence of immature hematopoietic progenitors in the spleen, as seen in our FLtg and FLtgxIL7tg mice, but not in LN (61). It appears that in FLtgxIL7tg mice, the expansion of both lymphoid and myeloid progenitors due to the combined action of both cytokines is sufficient to cause the accumulation of hematopoietic progenitors not only in the spleen but also in LN. This indicates that the environment in secondary lymphoid organs is able to support hematopoiesis in “emergency” situations, such as the one in FLtgxIL7tg mice, which manifest a pronounced myelo- and lympho-proliferative disease. However, it remains unknown whether this ability of the FLtgxIL7tg LN to support the accumulation of progenitors is due to alterations in the LN niche caused by high FL and IL-7 expression, e.g., up-regulated expression of other cytokines or adhesion molecules by LN stromal cells. Moreover, an interesting question is whether the FLtgxIL7tg LN is a site of on-going hematopoiesis, or if the progenitors only migrate there and accumulate without differentiating further. In support of the former hypothesis, FLtgxIL7tg LN-residing progenitors are functional in reconstituting hematopoietic cells after transfer to irradiated recipients (Figures 7, 8) and all hematopoietic developmental stages are represented in the LN in ratios similar to the ones found in WT BM. Further experiments would be needed to address this issue.

Collectively, our present analysis of FLtgxIL7tg mice demonstrates the *in vivo* synergistic action of FL and IL-7 in promoting lymphoid development and expansion. This is summarized in Supplementary Figure 9. Our data provide evidence that secondary lymphoid organs can support the maintenance of hematopoietic progenitors in conditions of abnormal hematopoiesis. Further studies of these mice might elucidate the requirements for extra-medullary residence and hematopoietic activity of HSC; an issue of clinical importance for the treatment of lympho-proliferative disorders and blood malignancies.

## Author contributions

FK, LvM, GC, SH, LA-S, HR, CE, MR, MM, GCv, and PT performed experiments. FK, PT, and AR analyzed data. PT wrote the manuscript. JA and RC contributed experimental ideas and revised the manuscript. PT and AR designed the study.

### Conflict of interest statement

The authors declare that the research was conducted in the absence of any commercial or financial relationships that could be construed as a potential conflict of interest.

## References

[1] Metcalf D. Hematopoietic cytokines. Blood (2008) 111:485–91. 10.1182/blood-2007-03-079681 18182579 PMC2200848

[2] Brown G Mooney CJ Alberti-Servera L Muenchow L Toellner KM Ceredig R. Versatility of stem and progenitor cells and the instructive actions of cytokines on hematopoiesis. Crit Rev Clin Lab Sci. (2015) 52:168–79. 10.3109/10408363.2015.1021412 26212176

[3] Brown G Tsapogas P Ceredig R. The changing face of hematopoiesis: a spectrum of options is available to stem cells. Immunol Cell Biol. (2018). 10.1111/imcb.12055. [Epub ahead of print]. 29637611

[4] Gilliland DG Griffin JD. The roles of FLT3 in hematopoiesis and leukemia. Blood (2002) 100:1532–42. 10.1182/blood-2002-02-0492 12176867

[5] Stirewalt DL Radich JP. The role of FLT3 in haematopoietic malignancies. Nat Rev Cancer (2003) 3:650–65. 10.1038/nrc1169 12951584

[6] Tsapogas P Mooney CJ Brown G Rolink A. The Cytokine Flt3-Ligand in Normal and Malignant Hematopoiesis. Int J Mol Sci. (2017) 18:E1115. 10.3390/ijms18061115 28538663 PMC5485939

[7] Adolfsson J Borge OJ Bryder D Theilgaard-Monch K Astrand-Grundstrom I Sitnicka E. Upregulation of Flt3 expression within the bone marrow Lin(-)Sca1(+)c-kit(+) stem cell compartment is accompanied by loss of self-renewal capacity. Immunity (2001) 15:659–69. 10.1016/S1074-7613(01)00220-5 11672547

[8] Mackarehtschian K Hardin JD Moore KA Boast S Goff SP Lemischka IR. Targeted disruption of the flk2/flt3 gene leads to deficiencies in primitive hematopoietic progenitors. Immunity (1995) 3:147–61. 7621074 10.1016/1074-7613(95)90167-1

[9] McKenna HJ Stocking KL Miller RE Brasel K De Smedt T Maraskovsky E. Mice lacking flt3 ligand have deficient hematopoiesis affecting hematopoietic progenitor cells, dendritic cells, and natural killer cells. Blood (2000) 95:3489–97. 10828034

[10] Buza-Vidas N Cheng M Duarte S Nozad H Jacobsen SE Sitnicka E. Crucial role of FLT3 ligand in immune reconstitution after bone marrow transplantation and high-dose chemotherapy. Blood (2007) 110:424–32. 10.1182/blood-2006-09-047480 17379745

[11] Holmes ML Carotta S Corcoran LM Nutt SL. Repression of Flt3 by Pax5 is crucial for B-cell lineage commitment. Genes Dev. (2006) 20:933–8. 10.1101/gad.1396206 16618805 PMC1472301

[12] Sitnicka E Bryder D Theilgaard-Monch K Buza-Vidas N Adolfsson J Jacobsen SE Key role of flt3 ligand in regulation of the common lymphoid progenitor but not in maintenance of the hematopoietic stem cell pool. Immunity (2002) 17:463–72. 10.1016/S1074-7613(02)00419-3 12387740

[13] von Muenchow L Alberti-Servera L Klein F Capoferri G Finke D Ceredig R. Permissive roles of cytokines interleukin-7 and Flt3 ligand in mouse B-cell lineage commitment. Proc Natl Acad Sci USA. (2016) 113:E8122–30. 10.1073/pnas.1613316113 27911806 PMC5167207

[14] Kondo M Weissman IL Akashi K. (1997). Identification of clonogenic common lymphoid progenitors in mouse bone marrow. Cell 91:661–72. 9393859 10.1016/s0092-8674(00)80453-5

[15] Namen AE Schmierer AE March CJ Overell RW Park LS Urdal DL. B cell precursor growth-promoting activity. Purification and characterization of a growth factor active on lymphocyte precursors. J Exp Med. (1988) 167:988–1002. 3258354 10.1084/jem.167.3.988 PMC2188872

[16] Rolink A Kudo A Karasuyama H Kikuchi Y Melchers F. Long-term proliferating early pre B cell lines and clones with the potential to develop to surface Ig-positive, mitogen reactive B cells *in vitro* and *in vivo*. EMBO J. (1991) 10:327–36. 1991449 10.1002/j.1460-2075.1991.tb07953.x PMC452650

[17] Peschon JJ Morrissey PJ Grabstein KH Ramsdell FJ Maraskovsky E Gliniak BC. Early lymphocyte expansion is severely impaired in interleukin 7 receptor-deficient mice. J Exp Med (1994) 180:1955–60. 7964471 10.1084/jem.180.5.1955 PMC2191751

[18] von Freeden-Jeffry U Vieira P Lucian LA McNeil T Burdach SE Murray R Lymphopenia in interleukin (IL)-7 gene-deleted mice identifies IL-7 as a nonredundant cytokine. J Exp Med (1995) 181:1519–26. 7699333 10.1084/jem.181.4.1519 PMC2191954

[19] Akashi K Kondo M von Freeden-Jeffry U Murray R Weissman IL. Bcl-2 rescues T lymphopoiesis in interleukin-7 receptor-deficient mice. Cell (1997) 89:1033–41. 9215626 10.1016/s0092-8674(00)80291-3

[20] Maraskovsky E O'Reilly LA Teepe M Corcoran LM Peschon JJ Strasser A Bcl-2 can rescue T lymphocyte development in interleukin-7 receptor-deficient mice but not in mutant rag-1–/– mice. Cell (1997) 89:1011–9. 9215624 10.1016/s0092-8674(00)80289-5

[21] Carrette F Surh CD. IL-7 signaling and CD127 receptor regulation in the control of T cell homeostasis. Semin Immunol. (2012) 24:209–17. 10.1016/j.smim.2012.04.010 22551764 PMC3367861

[22] Kondo M Akashi K Domen J Sugamura K Weissman IL. (1997). Bcl-2 rescues T lymphopoiesis, but not B or NK cell development, in common gamma chain-deficient mice. Immunity 7:155–62. 9252128 10.1016/s1074-7613(00)80518-x

[23] Maraskovsky E Peschon JJ McKenna H Teepe M Strasser A Overexpression of Bcl-2 does not rescue impaired B lymphopoiesis in IL-7 receptor-deficient mice but can enhance survival of mature B cells. Int Immunol. (1998) 10:1367–75. 9786436 10.1093/intimm/10.9.1367

[24] Dias S Silva H Jr Cumano A Vieira P. Interleukin-7 is necessary to maintain the B cell potential in common lymphoid progenitors. J Exp Med. (2005) 201:971–9. 10.1084/jem.20042393 15767371 PMC2213099

[25] Tsapogas P Zandi S Ahsberg J Zetterblad J Welinder E Jonsson JI. IL-7 mediates Ebf-1-dependent lineage restriction in early lymphoid progenitors. Blood (2011) 118:1283–90. 10.1182/blood-2011-01-332189 21652681

[26] Malin S McManus S Cobaleda C Novatchkova M Delogu A Bouillet P. Role of STAT5 in controlling cell survival and immunoglobulin gene recombination during pro-B cell development. Nat Immunol. (2010) 11:171–9. 10.1038/ni.1827 19946273 PMC2956121

[27] Fisher AG Burdet C LeMeur M Haasner D Gerber P Ceredig R. Lymphoproliferative disorders in an IL-7 transgenic mouse line. Leukemia (1993) 7(Suppl. 2):S66–8. 8361236

[28] Fisher AG Burdet C Bunce C Merkenschlager M Ceredig R. Lymphoproliferative disorders in IL-7 transgenic mice: expansion of immature B cells which retain macrophage potential. Int Immunol. (1995) 7:415–23. 7794821 10.1093/intimm/7.3.415

[29] Mertsching E Grawunder U Meyer V Rolink T Ceredig R. Phenotypic and functional analysis of B lymphopoiesis in interleukin-7-transgenic mice: expansion of pro/pre-B cell number and persistence of B lymphocyte development in lymph nodes and spleen. Eur J Immunol. (1996) 26:28–33. 10.1002/eji.1830260105 8566080

[30] Sitnicka E Brakebusch C Martensson IL Svensson M Agace WW Sigvardsson M. Complementary signaling through flt3 and interleukin-7 receptor alpha is indispensable for fetal and adult B cell genesis. J Exp Med. (2003) 198:1495–506. 10.1084/jem.20031152 14610045 PMC2194121

[31] Ahsberg J Tsapogas P Qian H Zetterblad J Zandi S Mansson R. Interleukin-7-induced Stat-5 acts in synergy with Flt-3 signaling to stimulate expansion of hematopoietic progenitor cells. J Biol Chem. (2010) 285:36275–84. 10.1074/jbc.M110.155531 20829349 PMC2978555

[32] Tsapogas P Swee LK Nusser A Nuber N Kreuzaler M Capoferri G. *In vivo* evidence for an instructive role of fms-like tyrosine kinase-3 (FLT3) ligand in hematopoietic development. Haematologica (2014) 99:638–46. 10.3324/haematol.2013.089482 24463214 PMC3971073

[33] Mazzucchelli R Durum SK. Interleukin-7 receptor expression: intelligent design. Nat Rev Immunol. (2007) 7:144–54. 10.1038/nri2023 17259970

[34] Ceredig R Rauch M Balciunaite G Rolink AG. Increasing Flt3L availability alters composition of a novel bone marrow lymphoid progenitor compartment. Blood (2006) 108:1216–22. 10.1182/blood-2005-10-006643 16675711

[35] Mertsching E Burdet C Ceredig R. IL-7 transgenic mice: analysis of the role of IL-7 in the differentiation of thymocytes *in vivo* and *in vitro*. Int Immunol. (1995) 7:401–14. 7794820 10.1093/intimm/7.3.401

[36] Swee LK Bosco N Malissen B Ceredig R Rolink A. Expansion of peripheral naturally occurring T regulatory cells by Fms-like tyrosine kinase 3 ligand treatment. Blood (2009) 113:6277–87. 10.1182/blood-2008-06-161026 19211508

[37] Carvalho TL Mota-Santos T Cumano A Demengeot J Vieira P. Arrested B lymphopoiesis and persistence of activated B cells in adult interleukin 7(-/)- mice. J Exp Med (2001) 194:1141–50. 10.1084/jem.194.8.1141 11602642 PMC2193519

[38] Jensen CT Kharazi S Boiers C Cheng M Lubking A Sitnicka E. FLT3 ligand and not TSLP is the key regulator of IL-7-independent B-1 and B-2 B lymphopoiesis. Blood (2008) 112:2297–304. 10.1182/blood-2008-04-150508 18566323

[39] Alberti-Servera L von Muenchow L Tsapogas P Capoferri G Eschbach K Beisel C. Single-cell RNA sequencing reveals developmental heterogeneity among early lymphoid progenitors. EMBO J (2017) 36:3619–33. 10.15252/embj.201797105 29030486 PMC5730887

[40] Balciunaite G Ceredig R Massa S Rolink AG. A B220+ CD117+ CD19- hematopoietic progenitor with potent lymphoid and myeloid developmental potential. Eur J Immunol. (2005) 35:2019–30. 10.1002/eji.200526318 15971276

[41] Kiel MJ Yilmaz OH Iwashita T Yilmaz OH Terhorst C Morrison SJ. SLAM family receptors distinguish hematopoietic stem and progenitor cells and reveal endothelial niches for stem cells. Cell (2005) 121:1109–21. 10.1016/j.cell.2005.05.026 15989959

[42] Pronk CJ Rossi DJ Mansson R Attema JL Norddahl GL Chan CK. Elucidation of the phenotypic, functional, and molecular topography of a myeloerythroid progenitor cell hierarchy. Cell Stem Cell (2007) 1:428–42. 10.1016/j.stem.2007.07.005 18371379

[43] Onai N Obata-Onai A Schmid MA Ohteki T Jarrossay D Manz MG. Identification of clonogenic common Flt3+M-CSFR+ plasmacytoid and conventional dendritic cell progenitors in mouse bone marrow. Nat Immunol. (2007) 8:1207–16. 10.1038/ni1518 17922016

[44] Liu K Victora GD Schwickert TA Guermonprez P Meredith MM Yao K. *In vivo* analysis of dendritic cell development and homeostasis. Science (2009) 324:392–7. 10.1126/science.1170540 19286519 PMC2803315

[45] Rodrigues PF Alberti-Servera L Eremin A Grajales-Reyes GE Ivanek R Tussiwand R Distinct progenitor lineages contribute to the heterogeneity of plasmacytoid dendritic cells. Nat Immunol. (2018) 19:711–22. 10.1038/s41590-018-0136-9 PMC7614340 29925996

[46] Tan JT Dudl E LeRoy E Murray R Sprent J Weinberg KI. IL-7 is critical for homeostatic proliferation and survival of naive T cells. Proc Natl Acad Sci USA. (2001) 98:8732–7. 10.1073/pnas.161126098 11447288 PMC37504

[47] Balciunaite G Ceredig R Rolink AG. The earliest subpopulation of mouse thymocytes contains potent T, significant macrophage, and natural killer cell but no B-lymphocyte potential. Blood (2005) 105:1930–6. 10.1182/blood-2004-08-3087 15522952

[48] Luc S Luis TC Boukarabila H Macaulay IC Buza-Vidas N Bouriez-Jones T. The earliest thymic T cell progenitors sustain B cell and myeloid lineage potential. Nat Immunol. (2012) 13:412–9. 10.1038/ni.2255 22344248 PMC3378629

[49] Mooney CJ Cunningham A Tsapogas P Toellner KM Brown G. Selective expression of Flt3 within the mouse hematopoietic stem cell compartment. Int J Mol Sci. (2017) 18:E1037. 10.3390/ijms18051037 28498310 PMC5454949

[50] Johns JL Christopher MM. Extramedullary hematopoiesis: a new look at the underlying stem cell niche, theories of development, and occurrence in animals. Vet Pathol. (2012) 49:508–23. 10.1177/0300985811432344 22262354

[51] Chiu SC Liu HH Chen CL Chen PR Liu MC Lin SZ. Extramedullary hematopoiesis (EMH) in laboratory animals: offering an insight into stem cell research. Cell Transplant (2015) 24:349–66. 10.3727/096368915X686850 25646951

[52] Sefc L Psenak O Sykora V Sulc K Necas E. Response of hematopoiesis to cyclophosphamide follows highly specific patterns in bone marrow and spleen. J Hematother Stem Cell Res. (2003) 12:47–61. 10.1089/152581603321210136 12662436

[53] Hsieh PP Olsen RJ O'Malley DP Konoplev SN Hussong JW Dunphy CH. The role of Janus Kinase 2 V617F mutation in extramedullary hematopoiesis of the spleen in neoplastic myeloid disorders. Mod Pathol. (2007) 20:929–35. 10.1038/modpathol.3800826 17643100

[54] Sasaki M Knobbe CB Munger JC Lind EF Brenner D Brustle A. IDH1(R132H) mutation increases murine haematopoietic progenitors and alters epigenetics. Nature (2012) 488:656–9. 10.1038/nature11323 22763442 PMC4005896

[55] Chang T Krisman K Theobald EH Xu J Akutagawa J Lauchle JO. Sustained MEK inhibition abrogates myeloproliferative disease in Nf1 mutant mice. J Clin Invest (2013) 123:335–9. 10.1172/JCI63193 23221337 PMC3533281

[56] Peters M Schirmacher P Goldschmitt J Odenthal M Peschel C Fattori E. Extramedullary expansion of hematopoietic progenitor cells in interleukin (IL)-6-sIL-6R double transgenic mice. J Exp Med. (1997) 185:755–66. 9034153 10.1084/jem.185.4.755 PMC2196150

[57] Khaldoyanidi S Sikora L Broide DH Rothenberg ME Sriramarao P. Constitutive overexpression of IL-5 induces extramedullary hematopoiesis in the spleen. Blood (2003) 101:863–8. 10.1182/blood-2002-03-0735 12393708

[58] Massberg S Schaerli P Knezevic-Maramica I Kollnberger M Tubo N Moseman EA. Immunosurveillance by hematopoietic progenitor cells trafficking through blood, lymph, and peripheral tissues. Cell (2007) 131:994–1008. 10.1016/j.cell.2007.09.047 18045540 PMC2330270

[59] Freud AG Becknell B Roychowdhury S Mao HC Ferketich AK Nuovo GJ. A human CD34(+) subset resides in lymph nodes and differentiates into CD56bright natural killer cells. Immunity (2005) 22:295–304. 10.1016/j.immuni.2005.01.013 15780987

[60] Maillard I Schwarz BA Sambandam A Fang T Shestova O Xu L. Notch-dependent T-lineage commitment occurs at extrathymic sites following bone marrow transplantation. Blood (2006) 107:3511–19. 10.1182/blood-2005-08-3454 16397133 PMC1895767

[61] Shurin MR Pandharipande PP Zorina TD Haluszczak C Subbotin VM Hunter O. FLT3 ligand induces the generation of functionally active dendritic cells in mice. Cell Immunol. (1997) 179:174–84. 10.1006/cimm.1997.1152 9268501

