# Supplementary material for: Accumulation of Multipotent Hematopoietic Progenitors in Peripheral Lymphoid Organs of Mice Over-expressing Interleukin-7 and Flt3-Ligand
Source: Front Immunol. 2018 Oct 10;9:2258. doi: 10.3389/fimmu.2018.02258 (PMC6191501; doi:10.3389/fimmu.2018.02258)
Supplement: Supplementary file 1 [file Data_Sheet_1.docx]

Supplementary Material

Accumulation of multipotent hematopoietic progenitors in peripheral lymphoid organs of mice over-expressing interleukin-7 and Flt3-ligand

**Fabian Klein^1^, Lilly von Muenchow^1^, Giuseppina Capoferri^1^, Stefan Heiler^1^, Llucia Alberti-Servera^1^, Hannie Rolink^1^, Corinne Engdahl^1^, Michael Rolink^1^, Mladen Mitrovic^1^, Grozdan Cvijetic^1^, Jan Andersson^1^, Rhodri Ceredig^2^, Panagiotis Tsapogas^1^*, Antonius Rolink^1^**

*** Correspondence:** Panagiotis Tsapogas: [panagiotis.tsapogas@unibas.ch](mailto:panagiotis.tsapogas@unibas.ch)

**
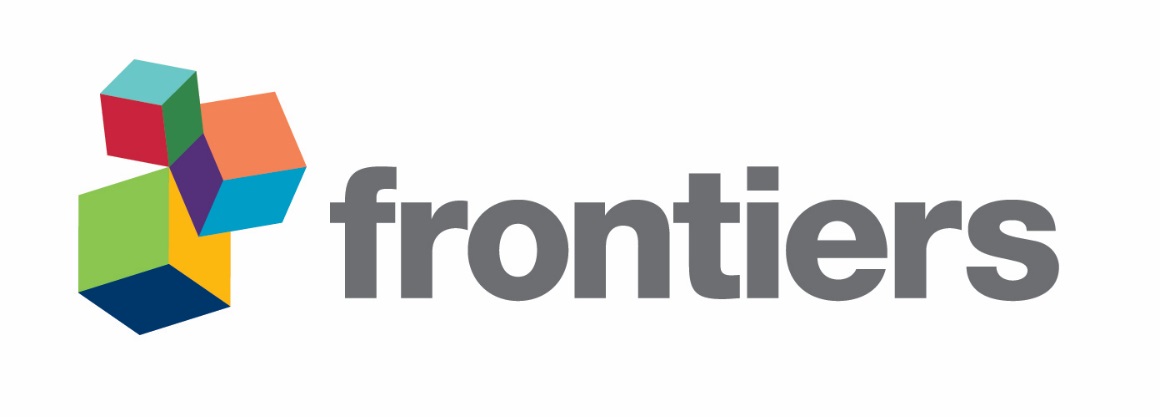
**

# Supplementary Figures

**Supplementary Figure 1**

**Myeloid and dendritic cells are increased in spleens and LN of FLtgxIL7tg mice.**

A) Numbers of myeloid cells in the spleen and lymph nodes (axillary and inguinal) of WT, FLtg, IL7tg and FLtgxIL7tg mice. Myeloid cells were identified as Gr1^+^CD11b^+^. B, D) Dendritic cell numbers in the spleen and lymph nodes of WT, FLtg, IL7tg and FLtgxIL7tg mice. C, E) Representative FACS plots for the identification of cDC1 (B220^-^SiglecH^-^MHC-II^+^CD11c^+^XCR1^+^), cDC2 (B220^-^SiglecH^-^MHC-II^+^CD11c^+^XCR1^-^) and pDC (B220^+^SiglecH^+^) in the spleen (C) and lymph nodes (E) of WT, FLtg, IL7tg and FLtgxIL7tg mice. Numbers on gates indicate percentage of parent gate. *: p<0.05, **:p<0.01,, ****:p<0.0001. Error bars indicate standard deviation.

**Supplementary Figure 2**

**T cell populations in FLtgxIL7tg mice**

A, B) Numbers of TCRβ^+^CD8^+^ (left panel), TCRβ^+^CD4^+^ (middle panel) and CD4^+^Foxp3^+^ (right panel) T cells in the spleens (A) and lymph nodes (axillary and inguinal) (B) of WT, FLtg, IL7tg and FLtgxIL7tg mice. C) Representative FACS plots for the identification of double negative (DN) CD4^-^CD8^-^ populations (DN1-4), double positive (DP; CD4^+^CD8^+^), CD4^+^ and CD8^+^ thymocytes in the thymi of WT, FLtg, IL7tg and FLtgxIL7tg mice. Lineage cocktail contained antibodies against: CD3, B220, CD11c and Gr1. Numbers on gates indicate percentage of parent gate. D) Numbers of DN, DP, CD4^+^ and CD8^+^ thymocytes in the thymi of WT, FLtg, IL7tg and FLtgxIL7tg mice. E) Numbers of DN1 (Lin^-^CD4^-^CD8^-^CD117^+^CD44^+^CD25^-^), DN2 (Lin^-^CD4^-^CD8^-^CD117^+^CD44^+^CD25^+^), DN3 (Lin^-^CD4^-^CD8^-^CD117^-^CD44^-^CD25^+^) and DN4 (Lin^-^CD4^-^CD8^-^CD117^-^CD44^-^CD25^-^) T cell progenitors in the thymi of WT, FLtg, IL7tg and FLtgxIL7tg mice. *: p<0.05, **:p<0.01, ***:p<0.001, ****:p<0.0001. Error bars indicate standard deviation.

**Supplementary Figure 3**

**B1 cells in the peritoneal cavity of FLtgxIL7tg mice.**

A) Representative FACS plots for the identification of B1a (CD19^+^CD11b^+^CD5^+^), B1b (CD19^+^CD11b^+^CD5^-^), B2 (CD19^+^CD11b^-^) and CD11b^+^ (CD19^-^CD11b^+^) myeloid cells in the peritoneal cavity of WT, FLtg, IL7tg and FLtgxIL7tg mice. Gate numbers indicate frequencies of parent gate. B) Percentages of B1a (left panel), B2 (middle panel) and myeloid cells (right panel) in the peritoneal cavity of WT, FLtg, IL7tg and FLtgxIL7tg mice. *: p<0.05, **:p<0.01, ***:p<0.001, ****:p<0.0001. Error bars indicate standard deviation.

**Supplementary Figure 4**

**Mature hematopoietic cell frequencies in peripheral blood of FLtgxIL7tg mice.**

Percentages of T cells (CD3^+^), B cells (CD19^+^), NK cells (CD3-NK1.1^+^), plasmacytoid DC (pDC; B220^+^SiglecH^+^), conventional DC (cDC; CD3^-^NK1.1^-^B220^-^CD11b^-^CD11c^+^) and myeloid cells (CD3^-^NK1.1^-^B220^-^CD11c^-^CD11b^+^) in the peripheral blood of WT, FLtg, IL7tg and FLtgxIL7tg mice (n=4 for all genotypes). *: p<0.05, **:p<0.01, ****:p<0.0001. Error bars indicate standard deviation.

**Supplementary Figure 5**

**Hematopoietic reconstitution potential of FLtgxIL7tg LN cells**.

A) Percentage of total CD45.2^+^ donor cells in spleen, bone marrow and thymus of mice reconstituted with FLtgxIL7tg BM (upper graph) or FLtgxIL7tg LN (lower graph). Black circles represent mice where the total donor contribution was scored as positive (>50 cells in the CD45.2^+^ gate). The fraction of positive-to-total mice analyzed for each organ is indicated above the corresponding bar. Results from four independently performed experiments are shown. Bars indicate mean ± standard error of the mean. B) Representative FACS plots indicating the total chimerism in the BM (upper plots), spleen (middle plots) and thymus (lower plots) of mice reconstituted with FLtgxIL7tg BM (left plots) and LN (right plots). C) Percentage of CD45.2^+^ donor cells within the indicated progenitor populations (LSK: Lin^-^CD127^-^CD117^+^Sca1^+^; CLP: Lin^-^CD117^int^Sca1^int^CD127^+^; proB: CD19^+^IgM^-^CD117^+^; large-preB: CD19^+^IgM^-^CD117^-^CD127^+^FSC^large^; small-preB: CD19^+^IgM^-^CD117^-^CD127^-^FSC^small^; and ImmB: CD19^+^IgM^+^CD93^+^) in the bone marrow of mice reconstituted with FLtgxIL7tg BM (up) or FLtgxIL7tg LN (down). Black circles represent mice where the corresponding cells were scored as positive for the presence of donor-derived cells (>50 cells in the CD45.2^+^ gate) and white circles mice with no reconstitution (<50 cells in the CD45.2^+^ gate). The ratio of positive-to-total mice analyzed for each progenitor population is indicated above the corresponding bar. Results from four independently performed experiments are shown. Bars indicate mean ± standard error of the mean. D) Representative FACS histograms showing the percentage of CD45.2^+^ FLtgxIL7tg BM- (left) and LN-derived (right) donor cells within the indicated progenitor populations of reconstituted mice.

**Supplementary Figure 6**

**Myelo-erythroid progenitors in the BM of FLtgxIL7tg mice.**

A) Representative FACS plots for the identification of pre-GM, GMP and pre-MegE progenitors in the BM of WT, FLtg, IL7tg and FLtgxIL7tg mice. Top FACS plots show cells that have been pre-gated as: live, CD117^+^Sca1^-^CD127^-^CD41^-^ cells. B) Numbers of pre-GM (left), GMP (middle) and pre-MegE (right) progenitors in the bone marrow of WT, FLtg, IL7tg and FLtgxIL7tg mice. Pre-GM: pre-granulocyte-macrophage progenitor (Lin^-^kit^+^Sca1^-^CD127^-^CD41^-^FcγRII/III^-^CD105^-^CD150^-^); GMP: granulocyte-macrophage progenitor (Lin^-^kit^+^Sca1^-^CD127^-^CD41^-^FcγRII/III^+^); pre-MegE: pre-megakaryocyte-erythroid progenitor (Lin^-^kit^+^Sca1^-^CD127^-^CD41^-^FcγRII/III^-^CD105^-^CD150^+^). *: p<0.05, **:p<0.01, ***:p<0.001, ****:p<0.0001. Bars indicate mean ± standard deviation.

**Supplementary Figure 7**

**Effect of FL and/or IL-7 over-expression on the cell-cycle status of BM and LN progenitors.**

A) Representative FACS histograms showing the percentage of Ki67^+^ cells within the indicated BM progenitor populations ((LSK: Lin^-^CD127^-^CD117^+^Sca1^+^; CLP: Lin^-^CD117^int^Sca1^int^CD127^+^; pro-B: CD19^+^IgM^-^CD117^+^; pre-B: CD19^+^IgM^-^CD117^-^ and IgM^+^CD93^+^) of WT, FLtg, IL7tg and FLtgxIL7tg mice. B) Frequencies of Ki67^+^ cells within the indicated BM progenitor populations of WT, FLtg, IL7tg and FLtgxIL7tg mice (n=4 for all genotypes). *: p<0.05, **:p<0.01, ***:p<0.001. Error bars indicate standard deviation. C) Representative FACS histograms showing the percentage of Ki67^+^ cells within the indicated LN progenitor populations of WT, FLtg, IL7tg and FLtgxIL7tg mice. D) Frequencies of Ki67^+^ cells within the indicated LN progenitor populations of WT, FLtg, IL7tg and FLtgxIL7tg mice (n=4 for all genotypes). *: p<0.05, **:p<0.01, ***:p<0.001, ****:p<0.0001. Error bars indicate standard deviation.

**Supplementary Figure 8**

**Expression of migratory-related proteins S1PR1, CD44 and CXCR4 on BM hematopoietic progenitors of FLtgxIL7tg mice.**

A) Representative FACS plots and histograms showing the percentages of S1PR1^+^, CXCR4^+^, S1PR1^+^CXCR4^+^ and CD44^+^ cells within the indicated BM progenitor populations (LSK: Lin^-^CD127^-^CD117^+^Sca1^+^; CLP: Lin^-^CD117^int^Sca1^int^CD127^+^; Ly6D^+^ EPLM: NK1.1^-^CD11c^-^SiglecH^-^CD19^-^B220^+^CD117^+^Ly6D^+^; pro-B: CD19^+^IgM^-^CD117^+^; pre-B: CD19^+^IgM^-^CD117^-^; and ImmB: CD19^+^IgM^+^CD93^+^) of WT, FLtg, IL7tg and FLtgxIL7tg mice. B) Percentages of S1PR1^+^, CXCR4^+^, S1PR1^+^CXCR4^+^ and CD44^+^ cells within the indicated BM progenitor populations of WT, FLtg, IL7tg and FLtgxIL7tg mice (n=4 for all genotypes). *: p<0.05, **:p<0.01, ***:p<0.001, ****:p<0.0001. Error bars indicate standard deviation.

**Supplementary Figure 9**

**Summary of the effect of increased FL and/or IL-7 levels in the numbers of hematopoietic progenitor and mature cells.**

Schematic representation of BM hematopoietic progenitors (open circles) and peripheral mature blood cells (grey cells). Arrows indicate developmental relationships. Blue circles indicate progenitors that express CD135 (Flt3) on their surface, red circles progenitors that express CD127 (IL7Rα) and purple circles progenitors that express both. Colored vertical arrows indicate an increase or decrease in the numbers of the corresponding cells in FLtg (blue arrows), IL7tg (red arrows) and FLtgxIL7tg mice (purple arrows). For progenitor cells the picture summarizes data from the BM and for mature cells from peripheral lymphoid organs. LSK: Lineage^-^Kit^+^Sca1^+^; GMP: Granulocyte-Macrophage Progenitor; MkP: Megakaryocyte Progenitor; ErP: Erythrocyte Progenitor; CLP: Common Lymphoid Progenitor; EPLM: Early Progenitor with Lymphoid and Myeloid potential; ETP: Early Thymic Progenitors, DN: CD4/CD8 Double-Negative, DP: CD4/CD8 Double-Positive.
