# Supplementary figures and images for: Accumulation of Multipotent Hematopoietic Progenitors in Peripheral Lymphoid Organs of Mice Over-expressing Interleukin-7 and Flt3-Ligand
Source: Front Immunol. 2018 Oct 10;9:2258. doi: 10.3389/fimmu.2018.02258 (PMC6191501; doi:10.3389/fimmu.2018.02258)

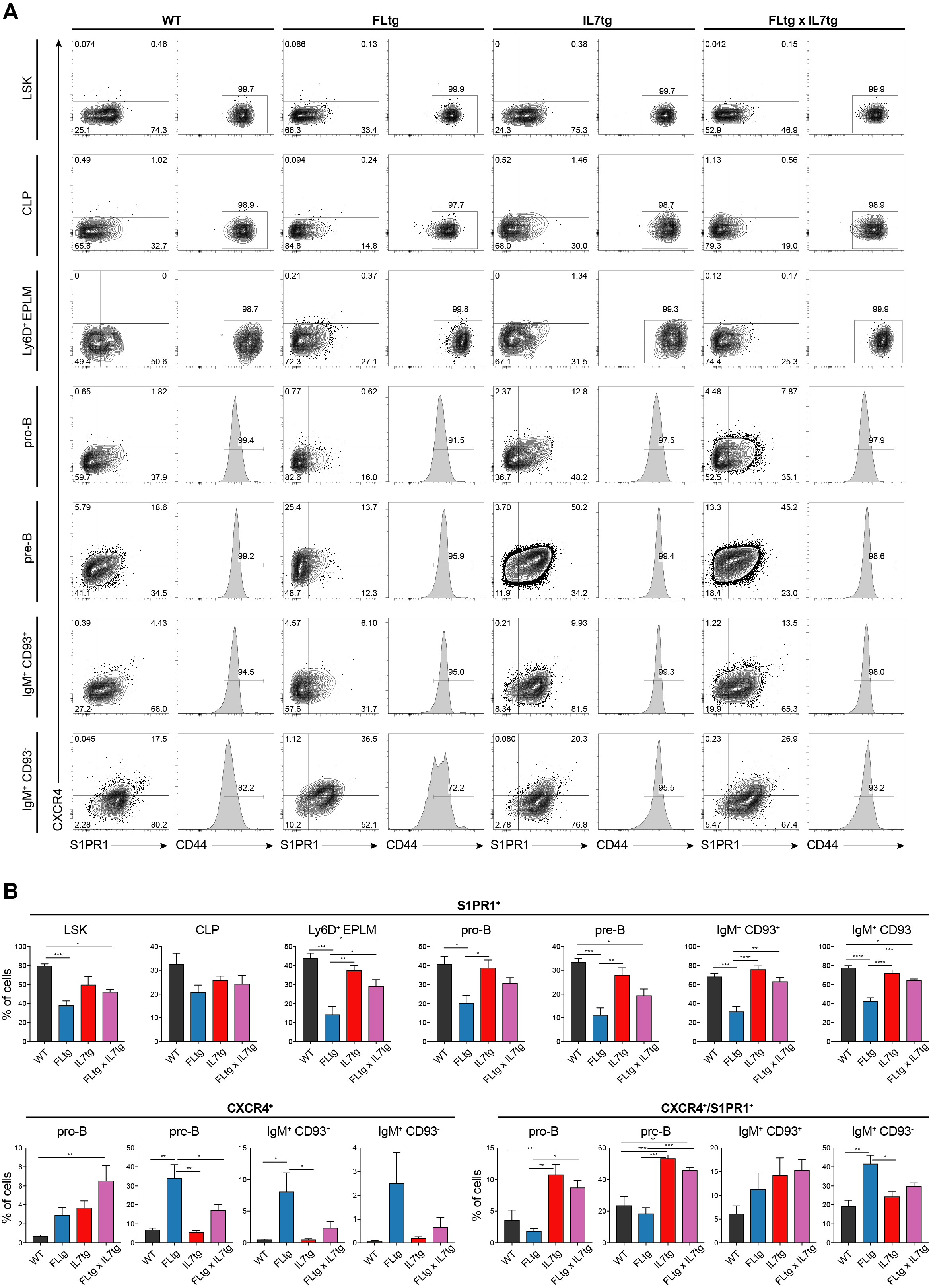

Supplement: Supplementary file 9 [file Image_8.png]
